# A Digital Health App to Assess Decisional Capacity to Provide Informed Consent: Protocol for a Randomized Controlled Trial

**DOI:** 10.2196/10360

**Published:** 2018-11-19

**Authors:** Robert D Furberg, Melissa Raspa, Anne C Wheeler, Lauren A McCormack, Donald B Bailey

**Affiliations:** 1 Digital Health & Clinical Informatics RTI International Research Triangle Park, NC United States; 2 Center for Newborn Screening, Ethics, and Disability Studies RTI International Research Triangle Park, NC United States; 3 Public Health Research Division RTI International Research Triangle Park, NC United States

**Keywords:** clinical trial, decision support techniques, digital health, ethics, informed consent, fragile X syndrome

## Abstract

**Background:**

Any study with human subjects must have a robust consent process to ensure that participants understand the study and can decide whether they want to be involved. Investigators must determine whether a potential study participant is able to make an informed decision and what modifications or supports are needed to maximize participation in decision making. A variety of approaches have been used to modify consent forms and the consent process to increase the research participants’ decisional capacity. This protocol describes a randomized controlled trial (RCT) of a digital health app to support decision making among individuals contemplating providing consent to participate in a clinical trial.

**Objective:**

The objective of this RCT will be to determine if the use of a tablet-based app facilitates greater participation in and satisfaction with the consent process compared with standard practice and identify which individual factors are associated with better response to the decision aid. We hypothesize that the tablet-based version of the consent process will promote more informed decision making, including decisions that are more consistent with individual preferences and values expressed during qualitative data collection.

**Methods:**

A two-arm RCT will be conducted in a sample of approximately 100 individuals with fragile X syndrome in their homes across the United States.

**Results:**

Data analysis will be completed by late 2018.

**Conclusions:**

By developing and testing a novel consent decision aid, we will have a better understanding of whether and how technological support can optimize the fit between the decisional capacity and the decisional process.

**Trial Registration:**

ClinicalTrials.gov NCT02465931; https://clinicaltrials.gov/ct2/show/NCT02465931 (Archived by WebCite at http://www.webcitation.org/72Q3xJQAw)

**International Registered Report Identifier (IRRID):**

PRR1-10.2196/10360

## Introduction

### Background

Any study with human subjects must have a robust consent process to ensure that participants understand the study and can decide whether they want to be involved. The Belmont Report [1] identified the following 3 ethical principles that should guide human subjects research: respect for persons, beneficence, and justice. However, investigators who study individuals with intellectual disability (ID) face a special challenge; they must determine whether a potential study participant is able to make an informed decision and what modifications or supports are needed to maximize participation in decision making. Fulfilling this obligation responsibly requires an understanding of the necessary components of consent, knowledge of common features of the cause of the person’s ID, and evidence-based adaptations to maximize informed decision making. Ultimately, ID researchers must acknowledge the delicate balance between respect for autonomy and the responsibility to protect vulnerable individuals [2]. Unfortunately, there is limited research on the decisional capacity of people with ID and the supports they need to consent to participate in research studies. This study will expand this knowledge base through an assessment of a digital health intervention for adolescents and adults with fragile X syndrome (FXS). FXS is an excellent prototype because of the wide range of cognitive abilities in affected individuals and the recent increase in clinical trials for new medications targeted at the core biology of FXS.

### Fragile X Syndrome

FXS is the most common known inherited cause of ID. Males typically have moderate ID, although impairment can range from mild to severe; females typically have mild ID, ranging from normal cognition to moderate impairment [3-5]. FXS is highly associated with a range of cooccurring conditions, the most common of which are attention problems and anxiety [6]. Longitudinal studies of FXS have shown deficits in areas such as sustained attention, response inhibition, working memory, and other executive functions likely to be associated with the capacity to consent [7-10]. Reading is challenging for males [11]. A large national survey found that although 44% of adult males with FXS could read basic picture books or simple stories, only 19% could read books that contain new words or concepts [12]. In contrast, 91% of adult females with FXS could read basic picture books or simple stories, and 76% could read books that contain new words or concepts. Further complicating these findings is the high coassociation between FXS and autism and consistent findings that individuals with both FXS and autism exhibit more severe deficits [9,11].

Recent advances in understanding the molecular basis of FXS have led to a new generation of targeted treatments [13,14], and clinical trials are under way using a variety of novel compounds. Owing to the possibility of side effects and the potential for significant changes in behavior or ability as a result of taking these medications, the importance of obtaining meaningful consent, not only from parents but also from individuals with FXS, has been elevated to a new level. Researchers and Institutional Review Board (IRB) members need data to guide decisions about involving individuals with FXS in the consent process. Unfortunately, little is known about the extent to which individuals with FXS can be or are involved in decisions about research participation.

### Decisional Capacity and Informed Consent

A variety of approaches have been used to modify consent forms and the consent process to increase the research participants’ decisional capacity. Early research focused on simplifying language (eg, shorter sentences and less technical vocabulary) and modifying the presentation (eg, bulleted or bolded text) [15-17]. Multimedia formats, such as slides, videos, or touchscreen computer programs, have also been tested [18-20]. Other approaches have sought to include a third party, such as a nurse or counselor, in the consent process [21,22]. Another alternative has focused on testing the participant on information contained in the consent form and providing feedback on incorrect answers [23,24].

However, most of these studies have not focused on individuals with ID. In a recent review, Goldsmith et al [25] summarized 22 studies of interventions designed specifically for individuals with ID. A primary finding was that life experiences—residence, history of decision making, and previous health experiences—contributed to the ability to provide consent [26-29]. Another key finding is that the method of presentation is important, especially for individuals with poor communication skills or lower memory ability [19,30]. Many studies have shown that general intelligence, verbal ability, and memory are correlated with the ability to consent [26-31].

Despite these conclusions, we still do not have a validated digital health app to enhance the decisional capacity in individuals with ID, and there is no consensus on the best approach to use [32]. A variety of techniques may be needed depending on the skill level of participants [33]. For example, although individuals with FXS have some weaknesses in visual-spatial processing [34], they have a relative strength in visual contextual memory [35], suggesting visual cues may help increase understanding. Recent papers suggested that the use of new technologies, primarily apps designed for tablets such as the iPad, have great potential for enhancing communication with people with ID [36,37]. Tablets are designed to be engaging and relatively intuitive to use. Our belief is that an appropriately designed tablet-based informed consent app has great potential for enhancing the decisional capacity.

This study will be the first systematic investigation of the decisional capacity in FXS in which we will develop and evaluate a decision aid (DA) with the intent of enhancing participation in the informed consent processes. The content of the digital informed consent app is based on an existing gold standard measure of the decisional capacity, the MacArthur Competence Assessment Tool for Clinical Research (MacCAT-CR) [38]. The MacCAT-CR is a structured clinical interview used to assess the capacity to consent in individuals with known or suspected deficits in cognitive ability. The interview covers the following 4 areas of the capacity to consent: understanding of the information presented about the nature of the research project; appreciation of the effects of research participation (or nonparticipation) on the potential participant’s own situation; reasoning about the decision to participate (or not); and expressing a choice about participation. The MacCAT-CR has been used in research settings with individuals with Alzheimer’s disease or schizophrenia [39,40]. Although the MacCAT-CR has been adapted for use in individuals with general ID [41], it has never been used to examine the capacity in a specific subpopulation, such as FXS, or been modified for use in a digital health app. Below we briefly describe the development of a tablet-based app.

### Description of the Digital Health App

**Figure 1:**
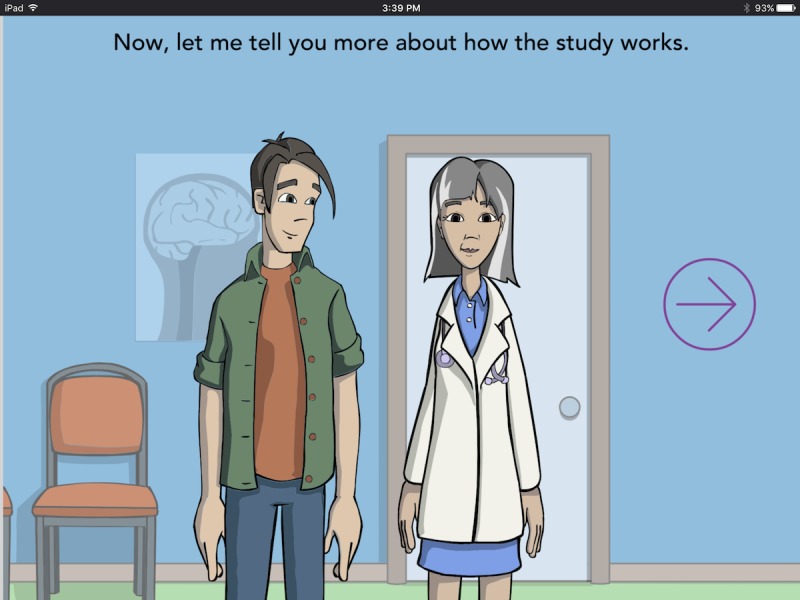
FXSDA screenshot.

**Figure 2:**
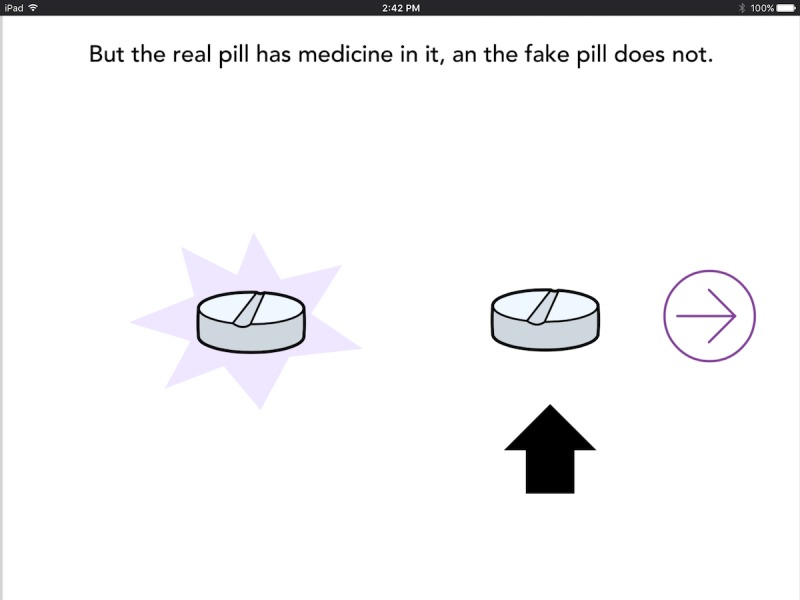
FXSDA screenshot.

Design and development of the intervention material and the principles that informed our approach are described elsewhere [42]. The FXS DA is a tablet-optimized, responsive Web app that delivers content through 3 major components as follows: scenario-based vignettes to present key concepts on clinical trials, informed consent, and other IRB-required material; quiz items to assess the decisional capacity based on the MacCAT-CR; and a tile sorting activity to provide a values clarification exercise at the conclusion of each session (Figures 1 and 2). Material for the DA was developed using input from a multidisciplinary committee of experienced developmental or clinical psychologists, clinicians, and communication scientists, based on data from qualitative interviews conducted with caregivers and individuals with FXS. Technical activities were informed by an agile, user-centered design approach, existing literature on the principles of universal design, and best practices for developing DA. The DA was implemented in Hypertext Markup Language 5 using CreateJS libraries for integration of animated multimedia, audio narration, and active tasks. The user interface and user experience were optimized for iOS tablet-based deployment and support interaction through standard touchscreen gestural controls (eg, swipe, tap, drag, and pinch).

A custom case management system enables event logging for each study participant, including case identifier, interaction specifics, and session time stamps. Built-in app analytics report events via JavaScript Object Notation packets sent over Secure Hypertext Transfer Protocol to a data management service that logs events, storing data in an encrypted relational database. Data extraction and reporting are performed by analysts using Structured Query Language queries and scripted transformations to prepare session data for the statistical analysis. FXS DA sessions lasted approximately 25 minutes and were intended to be deployed under supervised, experimental conditions in a study participant’s home or clinical setting by field staff. Devices used for field deployment were configured and conditioned throughout data collection in a manner consistent with the recommendations for tablet-based digital health intervention research by Furberg et al [43]. A video demonstration of the FXS DA is available in Multimedia Appendix 1.

### Research Questions and Hypotheses

The primary research question was, “Does a tablet-based app facilitate greater participation in and satisfaction with the consent process compared with standard practice?” We hypothesize that the tablet-based version of the consent process will promote more informed decision making, including decisions that are more consistent with individual preferences and values expressed during qualitative data collection. The secondary research question was, “What individual factors, such as IQ, autism status, or executive function, are associated with better response to the decision aid?” We hypothesize that all individuals with FXS will benefit from the tablet-based version, but those with higher functioning levels may benefit the most. Potential advantages of a tablet-based approach to patient education include consistent content and delivery, active learning, privacy, potentially greater access than a human health educator, and potential cost-effectiveness [44]. We expect that the interactive nature of the app will foster greater involvement in the decision making, a sense of empowerment, greater self-efficacy, and more satisfaction.

## Methods

### Trial Design

We will use a two-arm, parallel-design, randomized control trial (RCT) with a 1:1 allocation ratio. Participants will be randomized into either the control or experimental group. As shown in Table 1, both groups will be exposed to the same informed consent content but delivered through different channels—a digital informed consent app (experimental) or paper informed consent or usual practice (control). The content of both versions of the informed consent describes the requirements for participating in a hypothetical clinical trial for a fake prescription drug for FXS. Both versions include IRB-required information (eg, study procedures, study duration, and compensation). The digital informed consent app does not meet all of the regulated technical requirements for electronic informed consent (ie, the signature component). Additional details regarding trial design and conduct can be found in the CONSORT-EHEALTH checklist (Multimedia Appendix 2).

### Participants

Up to 50 pairs of adolescents or adults (aged ≥14 years) with FXS, a subset of 250 individuals who completed a battery of neurocognitive assessments (including intelligence quotient, reading abilities, autism, and anxiety comorbidities) between 2013 and 2016, will participate in this study.

**Table 1:** An overview of the study design, with details regarding how the variables differ across conditions.

Component	Control	Experimental
Delivery of information	Paper informed consent form paired with verbal overview of key points	Paper informed consent form paired with tablet-based tool which contains visual and audio components
Language	Complex (paper) and simplified (verbal overview)	Complex (paper) and simplified (tablet-based tool)
Exposure to information	Paper informed consent form will be sent to participant and family before data collection visit. They will be able to review as many times as they wish before visit During visits, simplified overview of informed consent form will be provided in person just once	Paper informed consent form will be sent to participant and family before data collection visit. They will be able to review as many times as they wish before visit During visit, the participant can go through the tablet-based tool up to 3 times
MacCAT questions	All questions will be asked after the disclosure information has been presented Questions will have the same wording as experimental condition Procedures will mimic MacCAT or flipchart (incorrect or partial credit will be given opportunity to answer question again after disclosure information is repeated) Multiple choice options rather than open-ended Paper and pencil data collection	All questions will be embedded within the vignettes or presentation of disclosure information Use simplified wording for questions- similar to flipchart Procedures will mimic MacCAT or flipchart (incorrect or partial credit will be given opportunity to answer question again after disclosure information is repeated) Multiple choice options rather than open-ended. Response data stored within tool and exported to dataset for analysis

### Recruitment

In this study, the following 3 primary methods will be used for recruitment: recontacting families who have participated in prior longitudinal assessment studies conducted by the research team; recruiting through the fragile X research registry maintained by the University of North Carolina at Chapel Hill; and announcing the research on the National Fragile X Foundation website. We have been highly successful in recruiting fragile X study participants, including a large national survey of >1000 families [45] so anticipate few problems in recruiting an adequate sample.

### Inclusion or Exclusion Criteria

Eligibility will be determined by a person’s scores on an initial assessment, which includes a standardized IQ and autism measure. Participants will be excluded if they receive a score of ≤30 on the IQ measure; receive a score between 31 and 40 on the IQ measure and have a diagnosis of autism with severe autism symptoms as noted on a standardized autism measure; or are determined to have other behavioral challenges that would preclude their inclusion (eg, mutism and severe aggression). These exclusion criteria cutoffs were established to ensure a minimal level of comprehension and adaptive behavior for the study; IQs of ≤35 are indicative of severe ID.

### Intervention

#### Control Group

Participants randomized to the control group will be exposed to a paper consent form that covers the same informed consent information presented in the tablet-based app and mimics informed consent forms used for real clinical trials. The complex language that is typically used in current standard practice (ie, *participation* vs *take part*) will be retained in the paper version. The control group will be designed to mimic typical informed consent procedures, that is, the verbal transmission of study information from a clinician to the individual and caregivers. To standardize this practice, we will develop a script that is verbally reviewed with control group participants, including a simplified overview of the key information in the paper consent form.

#### Experimental Group

Participants randomized to the experimental group will receive the paper consent form (the same form the control group participants received) and will also be exposed to the tablet-based app. Given that the digital app does not meet the requirements of electronic consent, the paper consent form will still be needed for documenting that informed consent was obtained.

### Randomization

We will use a stratified, block randomization method to assign participants to the control or experimental group. Two stratification variables will be used—verbal IQ score (3 levels) and age (2 levels). Age was selected as a randomizing variable because children and adolescents under 18 are not able to provide informed consent, only assent, as their parents are their legal guardians. Verbal IQ was selected as a second variable because we had a small sample size and wanted to control for any possible effects on the outcome variables. However, we could have also chosen to account for any possible group differences based on IQ through statistical analyses. Given that enrollment for the RCT will be done on a rolling basis, a 10-block, 2-group design will be used. Thus, we will randomize 10 participants at a time into either the control or experimental group. Furthermore, we will utilize a random number generator (www.randomizer.com) to make the assignments.

### Blinding

Owing to the nature of the study, participants and data collectors were not blinded to the group assignment.

### Study Setting and Data Collection Procedures

Each study session will occur in the individual’s home and will be videotaped to allow subsequent coding of individual engagement in the decisional process. Approximately 10 days prior to the visit, the participant or their primary caregiver will be sent the standard paper consent form for a hypothetical clinical trial. All participants, including parents and caregivers, will be informed that the clinical trial is hypothetical. Participants will be asked to review the consent form as they would any research consent form.

On the day of the visit, parents and individuals with FXS who are their own legal guardian will be asked to complete a 5- to 10-minute pretest to assess their or their child’s belief and attitudes about participating in clinical trials. Pretest items included questions such as participants’ possible reasons for participating in clinical trials, the likelihood of participating, and whom they think should make the decision about enrolling in a trial (full list of domains in “Study Outcomes” section below). Participants will then complete either the experimental or control group informed consent procedure. Both the control and experimental groups will be asked the modified MacCAT examination items throughout the informed consent process to assess the decisional capacity. The questions are identical for each group. After the informed consent procedure, all participants will again be asked to complete the posttest questions about their beliefs and attitudes about clinical trials.

### Study Outcomes

The following measures will be collected as part of the study protocol: time spent with DA or in discussion with research assistant; perceptions about reasons to participate in trials (eg, altruism; pre- and posttest item); likelihood of enrolling in a clinical trial (pre- and posttest item); the preferred level of involvement in the decision (pre- and posttest item); self-efficacy related to decision-making ability (pre- and posttest item); level of engagement in the decision-making process (pre- and posttest item); satisfaction with the decision (posttest only); the perceived value of the educational information provided (posttest only); and session analytics, including time on page and session duration.

### Participant Timeline

Pretest measures, the intervention or control condition, and posttest measures will all be conducted on the same day during an approximately 1-hour study session. The pre- and posttest measures will take approximately 15 minutes to complete, and the control and intervention condition will last approximately 30 minutes. No additional follow-up is planned.

### Statistical Methods

In the analyses, we will examine the effect of the app on the decisional capacity, controlling for sociodemographic characteristics and severity of delay. We will first conduct bivariate analyses comparing the decisional capacity, decision-making preferences, and the likelihood of trial participation across the 2 study groups (tablet vs standard procedure), using chi-square tests for categorical outcomes and *t* tests for continuous outcomes. In addition, we will conduct multiple regression models to compare study outcomes by the study group after controlling for demographics and severity of developmental delay. Linear regression models will be conducted for continuous outcomes (eg, decisional capacity scores and preferences) and logistic regression models for categorical outcomes (eg, the likelihood of participating in a hypothetical trial). Within these models, we will test for interactions between study group and demographic characteristics to identify the differential impact of the intervention on particular subgroups. For example, testing an interaction between study group and severity of delay would allow us to determine whether the tablet-based intervention is more or less successful among more impaired participants.

### Power Analyses

**Figure 3:**
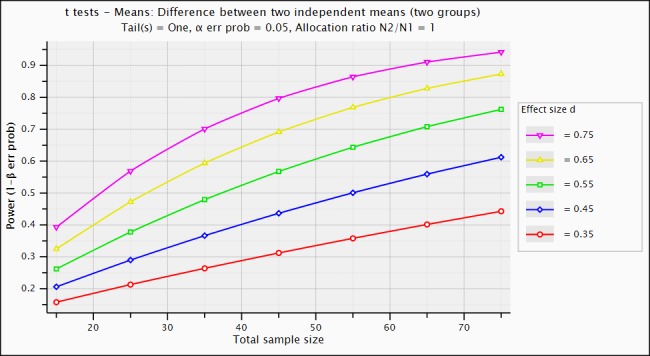
FXSDA screenshot.

We have conducted a power analysis using a between-subjects design (ie, participants are randomized to the control or experimental group) to determine the recommended sample size (Figure 3). With a sample size of 70 (35 participants per group), we will have 90% power to detect an effect size of 0.75.

### Ethics and Confidentiality

This research protocol was reviewed and approved by 2 Independent Review Boards from the University of North Carolina Office of Human Research Ethics (IRB Number: 13-1128) and RTI International Office of Research Protection (0281200.276). This study has been registered with ClinicalTrials.gov (NCT02465931).

## Results

Data collection began in January 2016 and concluded in January 2017. Data analysis began in mid-2017 and will be completed by late 2018.

## Discussion

### Principal Findings

Discoveries about the underlying mechanisms of conditions such as FXS inevitably will lead to a new generation of targeted pharmaceuticals to be tested in clinical trials. Consenting individuals with ID to participate in clinical trials will always require an individualized approach. But in-depth knowledge about the nature and range of the decisional capacity, cognitive and experiential factors influencing the decisional capacity, and the use of various DAs can make the individualization process more efficient and effective. We chose FXS as the condition of interest for this app because recent advances in treatment potential have led to a rapid growth in clinical trials testing new medications and the extraordinary range in cognitive ability and emotional problems in FXS, both within and across genders, makes it virtually impossible to characterize the decisional capacity of the population as a group. By describing and explaining the range of the decisional capacity in individuals with FXS, we will have a better estimate of both how many and how well individuals with FXS can participate in the consent process. By developing and testing a novel consent DA, we will have a better understanding of whether and how technological support can optimize the fit between the decisional capacity and the decisional process.

### Study Strengths

This protocol provides an overview of the design and implementation of a distributed, RCT to evaluate the use of a digital health app to support individual decision making for participation in clinical trials. Relatively few studies have been published on the use of digital resources to support this type of decision making given the ethical challenges of conducting such research without compromising the ethical or legal credibility and protections for human subjects.

### Limitations

Despite the strength of the evaluation design and scalability, the major limitation of this study is the focus on assessing decision making to participate in a hypothetical clinical trial.

## Appendix

Multimedia Appendix 1 FXSDA Parent Demo

Multimedia Appendix 2 CONSORT-EHEALTH checklist (V 1.6.1).

## Abbreviations

DA: Decision Aid
FXS: fragile X syndrome
ID: intellectual disability
IRB: Institutional Review Board
MacCAT: MacArthur Competence Assessment Tool for Clinical Research
RCT: randomized controlled trial
