# Supplementary material for: A Digital Health App to Assess Decisional Capacity to Provide Informed Consent: Protocol for a Randomized Controlled Trial
Source: JMIR Res Protoc. 2018 Nov 19;7(11):e10360. doi: 10.2196/10360 (PMC6277828; doi:10.2196/10360)
Supplement: Multimedia Appendix 2 [file resprot_v7i11e10360_app2.pdf]

CONSORT-EHEALTH (V 1.6.1) - Submission/Publication Form

The CONSORT-EHEALTH checklist is intended for authors of randomized trials evaluating web-based and Internet-based applications/interventions, including mobile interventions, electronic games (incl multiplayer games), social media, certain telehealth applications, and other interactive and/or networked electronic applications. Some of the items (e.g. all subitems under item 5 - description of the intervention) may also be applicable for other study designs.

The goal of the CONSORT EHEALTH checklist and guideline is to be
a) a guide for reporting for authors of RCTs,
b) to form a basis for appraisal of an ehealth trial (in terms of validity)

CONSORT-EHEALTH items/subitems are MANDATORY reporting items for studies published in the Journal of Medical Internet Research and other journals / scientific societies endorsing the checklist.

Items numbered 1., 2., 3., 4a., 4b etc are original CONSORT or CONSORT-NPT (non-pharmacologic treatment) items.
Items with Roman numerals (i., ii, iii, iv etc.) are CONSORT-EHEALTH extensions/clarifications.

As the CONSORT-EHEALTH checklist is still considered in a formative stage, we would ask that you also RATE ON A SCALE OF 1-5 how important/useful you feel each item is FOR THE PURPOSE OF THE CHECKLIST and reporting guideline (optional).

Mandatory reporting items are marked with a red *.
In the textboxes, either copy & paste the relevant sections from your manuscript into this form - please include any quotes from your manuscript in QUOTATION MARKS,
or answer directly by providing additional information not in the manuscript, or elaborating on why the item was not relevant for this study.

YOUR ANSWERS WILL BE PUBLISHED AS A SUPPLEMENTARY FILE TO YOUR PUBLICATION IN JMIR AND ARE CONSIDERED PART OF YOUR PUBLICATION (IF ACCEPTED).
Please fill in these questions diligently. Information will not be copyedited, so please use proper spelling and grammar, use correct capitalization, and avoid abbreviations.

DO NOT FORGET TO SAVE AS PDF _AND_ CLICK THE SUBMIT BUTTON SO YOUR ANSWERS ARE IN OUR DATABASE !!!

Citation Suggestion (if you append the pdf as Appendix we suggest to cite this paper in the caption):
Eysenbach G, CONSORT-EHEALTH Group
CONSORT-EHEALTH: Improving and Standardizing Evaluation Reports of Web-based and Mobile Health Interventions
J Med Internet Res 2011;13(4):e126
URL: http://www.jmir.org/2011/4/e126/
doi: 10.2196/jmir.1923
PMID: 22209829

* Required

Your name *

First Last

Robert Furberg

Primary Affiliation (short), City, Country *

University of Toronto, Toronto, Canada

RTI International

Your e-mail address *

abc@gmail.com

rfurberg@rti.org

Title of your manuscript *

Provide the (draft) title of your manuscript.

A Digital Health App to Assess Decisional Capacity to Provide Informed Consent: Randomized Controlled Trial Protocol

Name of your App/Software/Intervention *

If there is a short and a long/alternate name, write the short name first and add the long name in brackets.

Fragile X Syndrome Decision Aid (FXSDA)

Evaluated Version (if any)

e.g. "V1", "Release 2017-03-01", "Version 2.0.27913"

V1

Language(s) *

What language is the intervention/app in? If multiple languages are available, separate by comma (e.g. "English, French")

English

URL of your Intervention Website or App

e.g. a direct link to the mobile app on app in appstore (itunes, Google Play), or URL of the website. If the intervention is a DVD or hardware, you can also link to an Amazon page.

Your answer

URL of an image/screenshot (optional)

Your answer

Accessibility *

Can an enduser access the intervention presently?

- ○ access is free and open
- ○ access only for special usergroups, not open
- ○ access is open to everyone, but requires payment/subscription/in-app purchases
- ◉ app/intervention no longer accessible
- ○ Other:

Primary Medical Indication/Disease/Condition *

e.g. "Stress", "Diabetes", or define the target group in brackets after the condition, e.g. "Autism (Parents of children with)", "Alzheimers (Informal Caregivers of)"

Decision Aid supports review of informed

Primary Outcomes measured in trial *

comma-separated list of primary outcomes reported in the trial

Time spent using decision aid or in discus

Secondary/other outcomes

Are there any other outcomes the intervention is expected to affect?

Your answer

Recommended "Dose" *

What do the instructions for users say on how often the app should be used?

- ○ Approximately Daily
- ○ Approximately Weekly
- ○ Approximately Monthly
- ○ Approximately Yearly
- ◉ "as needed"
- ○ Other:

Approx. Percentage of Users (starters) still using the app as recommended after 3 months *

- (x) unknown / not evaluated
- ( ) 0-10%
- ( ) 11-20%
- ( ) 21-30%
- ( ) 31-40%
- ( ) 41-50%
- ( ) 51-60%
- ( ) 61-70%
- ( ) 71%-80%
- ( ) 81-90%
- ( ) 91-100%
- ( ) Other:

Overall, was the app/intervention effective? *

- ( ) yes: all primary outcomes were significantly better in intervention group vs control
- ( ) partly: SOME primary outcomes were significantly better in intervention group vs control
- ( ) no statistically significant difference between control and intervention
- ( ) potentially harmful: control was significantly better than intervention in one or more outcomes
- ( ) inconclusive: more research is needed
- (x) Other: Protocol paper, no results to report at this time

Article Preparation Status/Stage *

At which stage in your article preparation are you currently (at the time you fill in this form)

- ( ) not submitted yet - in early draft status
- ( ) not submitted yet - in late draft status, just before submission
- ( ) submitted to a journal but not reviewed yet
- ( ) submitted to a journal and after receiving initial reviewer comments
- (•) submitted to a journal and accepted, but not published yet
- ( ) published
- ( ) Other:

Journal *

If you already know where you will submit this paper (or if it is already submitted), please provide the journal name (if it is not JMIR, provide the journal name under "other")

- ( ) not submitted yet / unclear where I will submit this
- ( ) Journal of Medical Internet Research (JMIR)
- ( ) JMIR mHealth and UHealth
- ( ) JMIR Serious Games
- ( ) JMIR Mental Health
- ( ) JMIR Public Health
- ( ) JMIR Formative Research
- (•) Other JMIR sister journal
- ( ) Other:

Is this a full powered effectiveness trial or a pilot/feasibility trial? *

- ( ) Pilot/feasibility
- (•) Fully powered

Manuscript tracking number *

If this is a JMIR submission, please provide the manuscript tracking number under "other" (The ms tracking number can be found in the submission acknowledgement email, or when you login as author in JMIR. If the paper is already published in JMIR, then the ms tracking number is the four-digit number at the end of the DOI, to be found at the bottom of each published article in JMIR)

- ○ no ms number (yet) / not (yet) submitted to / published in JMIR
- ◉ Other: 10360

TITLE AND ABSTRACT

1a) TITLE: Identification as a randomized trial in the title

1a) Does your paper address CONSORT item 1a? *

I.e does the title contain the phrase "Randomized Controlled Trial"? (if not, explain the reason under "other")

- ◉ yes
- ○ Other:

1a-i) Identify the mode of delivery in the title

Identify the mode of delivery. Preferably use “web-based” and/or “mobile” and/or “electronic game” in the title. Avoid ambiguous terms like “online”, “virtual”, “interactive”. Use “Internet-based” only if Intervention includes non-web-based Internet components (e.g. email), use “computer-based” or “electronic” only if offline products are used. Use “virtual” only in the context of “virtual reality” (3-D worlds). Use “online” only in the context of “online support groups”. Complement or substitute product names with broader terms for the class of products (such as “mobile” or “smart phone” instead of “iphone”), especially if the application runs on different platforms.

	1	2	3	4	5	
subitem not at all important	○	○	○	○	◉	essential

Does your paper address subitem 1a-i? *

Copy and paste relevant sections from manuscript title (include quotes in quotation marks "like this" to indicate direct quotes from your manuscript), or elaborate on this item by providing additional information not in the ms, or briefly explain why the item is not applicable/relevant for your study

"A Digital Health App" to Assess Decisional Capacity to Provide Informed Consent: Randomized Controlled Trial Protocol

1a-ii) Non-web-based components or important co-interventions in title

Mention non-web-based components or important co-interventions in title, if any (e.g., “with telephone support”).

	1	2	3	4	5	
subitem not at all important	(●)	( )	( )	( )	( )	essential

Does your paper address subitem 1a-ii?

Copy and paste relevant sections from manuscript title (include quotes in quotation marks "like this" to indicate direct quotes from your manuscript), or elaborate on this item by providing additional information not in the ms, or briefly explain why the item is not applicable/relevant for your study

Non-web-based components or co-interventions were not used

1a-iii) Primary condition or target group in the title

Mention primary condition or target group in the title, if any (e.g., “for children with Type I Diabetes”)
Example: A Web-based and Mobile Intervention with Telephone Support for Children with Type I Diabetes: Randomized Controlled Trial

	1	2	3	4	5	
subitem not at all important	(●)	( )	( )	( )	( )	essential

Does your paper address subitem 1a-iii? *

Copy and paste relevant sections from manuscript title (include quotes in quotation marks "like this" to indicate direct quotes from your manuscript), or elaborate on this item by providing additional information not in the ms, or briefly explain why the item is not applicable/relevant for your study

Target audience

1b) ABSTRACT: Structured summary of trial design, methods, results, and conclusions

NPT extension: Description of experimental treatment, comparator, care providers, centers, and blinding status.

1b-i) Key features/functionalities/components of the intervention and comparator in the METHODS section of the ABSTRACT

Mention key features/functionalities/components of the intervention and comparator in the abstract. If possible, also mention theories and principles used for designing the site. Keep in mind the needs of systematic reviewers and indexers by including important synonyms. (Note: Only report in the abstract what the main paper is reporting. If this information is missing from the main body of text, consider adding it)

	1	2	3	4	5	
subitem not at all important	○	○	●	○	○	essential

Does your paper address subitem 1b-i? *

Copy and paste relevant sections from the manuscript abstract (include quotes in quotation marks "like this" to indicate direct quotes from your manuscript), or elaborate on this item by providing additional information not in the ms, or briefly explain why the item is not applicable/relevant for your study

"Objective: The objective of this RCT will be to determine if the use of a tablet-based app facilitates greater participation in and satisfaction with the consent process compared with standard practice and identify which individual factors are associated with better response to the decision aid. We hypothesize that the tablet-based version of the consent process will promote more informed decision making, including decisions that are more consistent with individual preferences and values expressed during qualitative data collection.
Methods: A 2-arm RCT will be conducted in a sample of approximately 100 individuals with fragile X syndrome in their homes across the United States.
Results: Data analysis will be completed in early 2018.
Conclusions: By developing and testing a novel consent decision aid, we will have a better understanding of whether and how technological support can optimize the fit between the decisional capacity and the decisional process."

1b-ii) Level of human involvement in the METHODS section of the ABSTRACT

Clarify the level of human involvement in the abstract, e.g., use phrases like “fully automated” vs. “therapist/nurse/care provider/physician-assisted” (mention number and expertise of providers involved, if any). (Note: Only report in the abstract what the main paper is reporting. If this information is missing from the main body of text, consider adding it)

	1	2	3	4	5	
subitem not at all important	○	○	◉	○	○	essential

Does your paper address subitem 1b-ii?

Copy and paste relevant sections from the manuscript abstract (include quotes in quotation marks "like this" to indicate direct quotes from your manuscript), or elaborate on this item by providing additional information not in the ms, or briefly explain why the item is not applicable/relevant for your study

"Objective: The objective of this RCT will be to determine if the use of a tablet-based app facilitates greater participation in and satisfaction with the consent process compared with standard practice and identify which individual factors are associated with better response to the decision aid. We hypothesize that the tablet-based version of the consent process will promote more informed decision making, including decisions that are more consistent with individual preferences and values expressed during qualitative data collection.
Methods: A 2-arm RCT will be conducted in a sample of approximately 100 individuals with fragile X syndrome in their homes across the United States.
Results: Data analysis will be completed in early 2018.
Conclusions: By developing and testing a novel consent decision aid, we will have a better understanding of whether and how technological support can optimize the fit between the decisional capacity and the decisional process."

1b-iii) Open vs. closed, web-based (self-assessment) vs. face-to-face assessments in the METHODS section of the ABSTRACT

Mention how participants were recruited (online vs. offline), e.g., from an open access website or from a clinic or a closed online user group (closed usergroup trial), and clarify if this was a purely web-based trial, or there were face-to-face components (as part of the intervention or for assessment). Clearly say if outcomes were self-assessed through questionnaires (as common in web-based trials). Note: In traditional offline trials, an open trial (open-label trial) is a type of clinical trial in which both the researchers and participants know which treatment is being administered. To avoid confusion, use “blinded” or “unblinded” to indicated the level of blinding instead of “open”, as “open” in web-based trials usually refers to “open access” (i.e. participants can self-enrol). (Note: Only report in the abstract what the main paper is reporting. If this information is missing from the main body of text, consider adding it)

	1	2	3	4	5	
subitem not at all important	○	○	◉	○	○	essential

Does your paper address subitem 1b-iii?

Copy and paste relevant sections from the manuscript abstract (include quotes in quotation marks "like this" to indicate direct quotes from your manuscript), or elaborate on this item by providing additional information not in the ms, or briefly explain why the item is not applicable/relevant for your study

"Objective: The objective of this RCT will be to determine if the use of a tablet-based app facilitates greater participation in and satisfaction with the consent process compared with standard practice and identify which individual factors are associated with better response to the decision aid. We hypothesize that the tablet-based version of the consent process will promote more informed decision making, including decisions that are more consistent with individual preferences and values expressed during qualitative data collection.
Methods: A 2-arm RCT will be conducted in a sample of approximately 100 individuals with fragile X syndrome in their homes across the United States.
Results: Data analysis will be completed in early 2018.
Conclusions: By developing and testing a novel consent decision aid, we will have a better understanding of whether and how technological support can optimize the fit between the decisional capacity and the decisional process."

1b-iv) RESULTS section in abstract must contain use data

Report number of participants enrolled/assessed in each group, the use/uptake of the intervention (e.g., attrition/adherence metrics, use over time, number of logins etc.), in addition to primary/secondary outcomes. (Note: Only report in the abstract what the main paper is reporting. If this information is missing from the main body of text, consider adding it)

	1	2	3	4	5	
subitem not at all important	●	○	○	○	○	essential

Does your paper address subitem 1b-iv?

Copy and paste relevant sections from the manuscript abstract (include quotes in quotation marks "like this" to indicate direct quotes from your manuscript), or elaborate on this item by providing additional information not in the ms, or briefly explain why the item is not applicable/relevant for your study

Protocol paper does not include any data at this time

1b-v) CONCLUSIONS/DISCUSSION in abstract for negative trials

Conclusions/Discussions in abstract for negative trials: Discuss the primary outcome - if the trial is negative (primary outcome not changed), and the intervention was not used, discuss whether negative results are attributable to lack of uptake and discuss reasons. (Note: Only report in the abstract what the main paper is reporting. If this information is missing from the main body of text, consider adding it)

	1	2	3	4	5	
subitem not at all important	○	○	●	○	○	essential

Does your paper address subitem 1b-v?

Copy and paste relevant sections from the manuscript abstract (include quotes in quotation marks "like this" to indicate direct quotes from your manuscript), or elaborate on this item by providing additional information not in the ms, or briefly explain why the item is not applicable/relevant for your study

"Objective: The objective of this RCT will be to determine if the use of a tablet-based app facilitates greater participation in and satisfaction with the consent process compared with standard practice and identify which individual factors are associated with better response to the decision aid. We hypothesize that the tablet-based version of the consent process will promote more informed decision making, including decisions that are more consistent with individual preferences and values expressed during qualitative data collection.
Methods: A 2-arm RCT will be conducted in a sample of approximately 100 individuals with fragile X syndrome in their homes across the United States.
Results: Data analysis will be completed in early 2018.
Conclusions: By developing and testing a novel consent decision aid, we will have a better understanding of whether and how technological support can optimize the fit between the decisional capacity and the decisional process."

INTRODUCTION

2a) In INTRODUCTION: Scientific background and explanation of rationale

2a-i) Problem and the type of system/solution

Describe the problem and the type of system/solution that is object of the study: intended as stand-alone intervention vs. incorporated in broader health care program? Intended for a particular patient population? Goals of the intervention, e.g., being more cost-effective to other interventions, replace or complement other solutions? (Note: Details about the intervention are provided in “Methods” under 5)

	1	2	3	4	5	
subitem not at all important	○	○	○	○	◉	essential

Does your paper address subitem 2a-i? *

Copy and paste relevant sections from the manuscript (include quotes in quotation marks "like this" to indicate direct quotes from your manuscript), or elaborate on this item by providing additional information not in the ms, or briefly explain why the item is not applicable/relevant for your study

"This study will be the first systematic investigation of decisional capacity in FXS in which we develop and evaluate a decision aid with the intent of enhancing participation in the informed consent processes. The content of the digital informed consent app is based on an existing gold standard measure of the decisional capacity, the MacArthur Competence Assessment Tool for Clinical Research (MacCAT-CR) [38]. The MacCAT-CR is a structured clinical interview used to assess the capacity to consent in individuals with known or suspected deficits in cognitive ability. The interview covers the following 4 areas of the capacity to consent: understanding of the information presented about the nature of the research project; appreciation of the effects of research participation (or nonparticipation) on the potential participant's own situation; reasoning about the decision to participate (or not); and expressing a choice about participation. The MacCAT-CR has been used in research settings with individuals with Alzheimer's disease or schizophrenia [39,40]. Although the MacCAT-CR has been adapted for use in individuals with general ID [41], it has never been used to examine the capacity in a specific subpopulation, such as FXS, or been modified for use in a digital health app. Below we briefly describe the development of a tablet-based app."

2a-ii) Scientific background, rationale: What is known about the (type of) system

Scientific background, rationale: What is known about the (type of) system that is the object of the study (be sure to discuss the use of similar systems for other conditions/diagnoses, if appropiate), motivation for the study, i.e. what are the reasons for and what is the context for this specific study, from which stakeholder viewpoint is the study performed, potential impact of findings [2]. Briefly justify the choice of the comparator.

	1	2	3	4	5	
subitem not at all important	○	○	◉	○	○	essential

Does your paper address subitem 2a-ii? *

Copy and paste relevant sections from the manuscript (include quotes in quotation marks "like this" to indicate direct quotes from your manuscript), or elaborate on this item by providing additional information not in the ms, or briefly explain why the item is not applicable/relevant for your study

"A variety of approaches have been used to modify consent forms and the consent process to increase the research participants' decisional capacity. Early research focused on simplifying language (eg, shorter sentences and less technical vocabulary) and modifying the presentation (eg, bulleted or bolded text) [15-17]. Multimedia formats, such as slides, videos, or touchscreen computer programs, have also been tested [18-20]. Other approaches have sought to include a third party, such as a nurse or counselor, in the consent process [21,22]. Another alternative has focused on testing the participant on information contained in the consent form and providing feedback on incorrect answers [23,24].
However, most of these studies have not focused on individuals with ID. In a recent review, Goldsmith et al [25] summarized 22 studies of interventions designed specifically for individuals with ID. A primary finding was that life experiences—residence, history of decision making, and previous health experiences—contributed to the ability to provide consent [26-29]. Another key finding is that the method of presentation is important, especially for individuals with poor communication skills or lower memory ability [19,30]. Many studies have shown that general intelligence, verbal ability, and memory are correlated with the ability to consent [26-31]."

2b) In INTRODUCTION: Specific objectives or hypotheses

Does your paper address CONSORT subitem 2b? *

Copy and paste relevant sections from the manuscript (include quotes in quotation marks "like this" to indicate direct quotes from your manuscript), or elaborate on this item by providing additional information not in the ms, or briefly explain why the item is not applicable/relevant for your study

"The primary research question was, “Does a tablet-based app facilitate greater participation in and satisfaction with the consent process compared with standard practice?” We hypothesize that the tablet-based version of the consent process will promote more informed decision making, including decisions that are more consistent with individual preferences and values expressed during qualitative data collection. The secondary research question was, “What individual factors, such as IQ, autism status, or executive function, are associated with better response to the decision aid?” We hypothesize that all individuals with FXS will benefit from the tablet-based version, but those with higher functioning levels may benefit the most. Potential advantages of a tablet-based approach to patient education include consistent content and delivery, active learning, privacy, potentially greater access than a human health educator, and potential cost-effectiveness [44]. We expect that the interactive nature of the app will foster greater involvement in the decision making, a sense of empowerment, greater self-efficacy, and more satisfaction."

METHODS

3a) Description of trial design (such as parallel, factorial) including allocation ratio

Does your paper address CONSORT subitem 3a? *

Copy and paste relevant sections from the manuscript (include quotes in quotation marks "like this" to indicate direct quotes from your manuscript), or elaborate on this item by providing additional information not in the ms, or briefly explain why the item is not applicable/relevant for your study

"We will use a 2-arm, parallel-design, randomized control trial (RCT) with a 1:1 allocation ratio. Participants will be randomized into either the control or experimental group. As shown in Table 1, both groups will be exposed to the same informed consent content but delivered through different channels—a digital informed consent app (experimental) or paper informed consent or usual practice (control). The content of both versions of the informed consent describes the requirements for participating in a hypothetical clinical trial for a fake prescription drug for FXS. Both versions include IRB-required information (eg, study procedures, study duration, and compensation). The digital informed consent app does not meet all of the regulated technical requirements for electronic informed consent (ie, the signature component)."

3b) Important changes to methods after trial commencement (such as eligibility criteria), with reasons

Does your paper address CONSORT subitem 3b? *

Copy and paste relevant sections from the manuscript (include quotes in quotation marks "like this" to indicate direct quotes from your manuscript), or elaborate on this item by providing additional information not in the ms, or briefly explain why the item is not applicable/relevant for your study

There were no changes to eligibility criteria after initiation of the trial

3b-i) Bug fixes, Downtimes, Content Changes

Bug fixes, Downtimes, Content Changes: ehealth systems are often dynamic systems. A description of changes to methods therefore also includes important changes made on the intervention or comparator during the trial (e.g., major bug fixes or changes in the functionality or content) (5-iii) and other “unexpected events” that may have influenced study design such as staff changes, system failures/downtimes, etc. [2].

	1	2	3	4	5	
subitem not at all important	○	○	◉	○	○	essential

Does your paper address subitem 3b-i?

Copy and paste relevant sections from the manuscript (include quotes in quotation marks "like this" to indicate direct quotes from your manuscript), or elaborate on this item by providing additional information not in the ms, or briefly explain why the item is not applicable/relevant for your study

There were no changes, fixes, or downtime events during the trial.

4a) Eligibility criteria for participants

Does your paper address CONSORT subitem 4a? *

Copy and paste relevant sections from the manuscript (include quotes in quotation marks "like this" to indicate direct quotes from your manuscript), or elaborate on this item by providing additional information not in the ms, or briefly explain why the item is not applicable/relevant for your study

"Eligibility will be determined by a person’s scores on an initial assessment, which includes a standardized IQ and autism measure. Participants will be excluded if they receive a score of □30 on the IQ measure; receive a score between 31 and 40 on the IQ measure and have a diagnosis of autism with severe autism symptoms as noted on a standardized autism measure; or are determined to have other behavioral challenges that would preclude their inclusion (eg, mutism and severe aggression). These exclusion criteria cutoffs were established to ensure a minimal level of comprehension and adaptive behavior for the study; IQs of □35 are indicative of severe ID."

4a-i) Computer / Internet literacy

Computer / Internet literacy is often an implicit “de facto” eligibility criterion - this should be explicitly clarified.

	1	2	3	4	5	
subitem not at all important	○	○	◉	○	○	essential

Does your paper address subitem 4a-i?

Copy and paste relevant sections from the manuscript (include quotes in quotation marks "like this" to indicate direct quotes from your manuscript), or elaborate on this item by providing additional information not in the ms, or briefly explain why the item is not applicable/relevant for your study

Digital literacy at baseline was not a consideration during the trial.

4a-ii) Open vs. closed, web-based vs. face-to-face assessments:

Open vs. closed, web-based vs. face-to-face assessments: Mention how participants were recruited (online vs. offline), e.g., from an open access website or from a clinic, and clarify if this was a purely web-based trial, or there were face-to-face components (as part of the intervention or for assessment), i.e., to what degree got the study team to know the participant. In online-only trials, clarify if participants were quasi-anonymous and whether having multiple identities was possible or whether technical or logistical measures (e.g., cookies, email confirmation, phone calls) were used to detect/prevent these.

	1	2	3	4	5	
subitem not at all important	○	○	◉	○	○	essential

Does your paper address subitem 4a-ii? *

Copy and paste relevant sections from the manuscript (include quotes in quotation marks "like this" to indicate direct quotes from your manuscript), or elaborate on this item by providing additional information not in the ms, or briefly explain why the item is not applicable/relevant for your study

"Each study session will occur in the individual's home and will be videotaped to allow subsequent coding of individual engagement in the decisional process. Approximately 10 days prior to the visit, the participant or their primary caregiver will be sent the standard paper consent form for a hypothetical clinical trial. All participants, including parents and caregivers, will be informed that the clinical trial is hypothetical. Participants will be asked to review the consent form as they would any research consent form."

4a-iii) Information giving during recruitment

Information given during recruitment. Specify how participants were briefed for recruitment and in the informed consent procedures (e.g., publish the informed consent documentation as appendix, see also item X26), as this information may have an effect on user self-selection, user expectation and may also bias results.

	1	2	3	4	5	
subitem not at all important	○	○	◉	○	○	essential

Does your paper address subitem 4a-iii?

Copy and paste relevant sections from the manuscript (include quotes in quotation marks "like this" to indicate direct quotes from your manuscript), or elaborate on this item by providing additional information not in the ms, or briefly explain why the item is not applicable/relevant for your study

"In this study, the following 3 primary methods will be used for recruitment: recontacting families who have participated in prior longitudinal assessment studies conducted by the research team; recruiting through the FX research registry maintained by the University of North Carolina at Chapel Hill; and announcing the research on the National Fragile X Foundation website. We have been highly successful in recruiting FX study participants, including a large national survey of >1000 families [45] so anticipate few problems in recruiting an adequate sample."

4b) Settings and locations where the data were collected

Does your paper address CONSORT subitem 4b? *

Copy and paste relevant sections from the manuscript (include quotes in quotation marks "like this" to indicate direct quotes from your manuscript), or elaborate on this item by providing additional information not in the ms, or briefly explain why the item is not applicable/relevant for your study

"Each study session will occur in the individual's home and will be videotaped to allow subsequent coding of individual engagement in the decisional process. Approximately 10 days prior to the visit, the participant or their primary caregiver will be sent the standard paper consent form for a hypothetical clinical trial. All participants, including parents and caregivers, will be informed that the clinical trial is hypothetical. Participants will be asked to review the consent form as they would any research consent form."

4b-i) Report if outcomes were (self-)assessed through online questionnaires

Clearly report if outcomes were (self-)assessed through online questionnaires (as common in web-based trials) or otherwise.

	1	2	3	4	5	
subitem not at all important	○	○	◉	○	○	essential

Does your paper address subitem 4b-i? *

Copy and paste relevant sections from the manuscript (include quotes in quotation marks "like this" to indicate direct quotes from your manuscript), or elaborate on this item by providing additional information not in the ms, or briefly explain why the item is not applicable/relevant for your study

"On the day of the visit, parents and individuals with FXS who are their own legal guardian will be asked to complete a 5- to 10-minute pretest to assess their or their child's belief and attitudes about participating in clinical trials. Pretest items included questions such as participants' possible reasons for participating in clinical trials, the likelihood of participating, and whom they think should make the decision about enrolling in a trial (full list of domains in "Study Outcomes" section below). Participants will then complete either the experimental or control group informed consent procedure. Both the control and experimental groups will be asked the modified MacCAT examination items throughout the informed consent process to assess the decisional capacity. The questions are identical for each group. After the informed consent procedure, all participants will again be asked to complete the posttest questions about their beliefs and attitudes about clinical trials."

4b-ii) Report how institutional affiliations are displayed

Report how institutional affiliations are displayed to potential participants [on ehealth media], as affiliations with prestigious hospitals or universities may affect volunteer rates, use, and reactions with regards to an intervention.(Not a required item – describe only if this may bias results)

	1	2	3	4	5	
subitem not at all important	●	○	○	○	○	essential

Does your paper address subitem 4b-ii?

Copy and paste relevant sections from the manuscript (include quotes in quotation marks "like this" to indicate direct quotes from your manuscript), or elaborate on this item by providing additional information not in the ms, or briefly explain why the item is not applicable/relevant for your study

Institutional affiliations are not displayed in the intervention material.

5) The interventions for each group with sufficient details to allow replication, including how and when they were actually administered

5-i) Mention names, credential, affiliations of the developers, sponsors, and owners

Mention names, credential, affiliations of the developers, sponsors, and owners [6] (if authors/evaluators are owners or developer of the software, this needs to be declared in a "Conflict of interest" section or mentioned elsewhere in the manuscript).

	1	2	3	4	5	
subitem not at all important	○	○	●	○	○	essential

Does your paper address subitem 5-i?

Copy and paste relevant sections from the manuscript (include quotes in quotation marks "like this" to indicate direct quotes from your manuscript), or elaborate on this item by providing additional information not in the ms, or briefly explain why the item is not applicable/relevant for your study

"Design and development of the intervention material and the principles that informed our approach are described elsewhere [42]. The FXS Decision Aid is a tablet-optimized, responsive Web app that delivers content through 3 major components as follows: scenario-based vignettes to present key concepts on clinical trials, informed consent, and other IRB-required material; quiz items to assess the decisional capacity based on the MacCAT-CR; and a tile sorting activity to provide a values clarification exercise at the conclusion of each session. Material for the Decision Aid was developed using input from a multidisciplinary committee of experienced developmental or clinical psychologists, clinicians, and communication scientists, based on data from qualitative interviews conducted with caregivers and individuals with FXS. Technical activities were informed by an agile, user-centered design approach, existing literature on the principles of universal design, and best practices for developing decision aids. The DA was implemented in Hypertext Markup Language 5 using CreateJS libraries for integration of animated multimedia, audio narration, and active tasks. The user interface and user experience were optimized for iOS tablet-based deployment and support interaction through standard touchscreen gestural controls (eg, swipe, tap, drag, and pinch).
A custom case management system enables event logging for each study participant, including case identifier, interaction specifics, and session time stamps. Built-in app analytics report events via JavaScript Object Notation packets sent over Secure Hypertext Transfer Protocol to a data management service that logs events, storing data in an encrypted relational database. Data extraction and reporting are performed by analysts using Structured Query Language queries and scripted transformations to prepare session data for the statistical analysis. FXS Decision Aid sessions lasted approximately 25 minutes and were intended to be deployed under supervised, experimental conditions in a study participant’s home or clinical setting by field staff. Devices used for field deployment were configured and conditioned throughout data collection in a manner consistent with the recommendations for tablet-based digital health intervention research by Furberg et al [43]. A video demonstration of the FXS Decision Aid is available in Multimedia Appendix 1."

5-ii) Describe the history/development process

Describe the history/development process of the application and previous formative evaluations (e.g., focus groups, usability testing), as these will have an impact on adoption/use rates and help with interpreting results.

	1	2	3	4	5	
subitem not at all important	(●)	( )	( )	( )	( )	essential

Does your paper address subitem 5-ii?

Copy and paste relevant sections from the manuscript (include quotes in quotation marks "like this" to indicate direct quotes from your manuscript), or elaborate on this item by providing additional information not in the ms, or briefly explain why the item is not applicable/relevant for your study

Design and development of the intervention material and the principles that informed our approach are described elsewhere [42]. The FXS Decision Aid is a tablet-optimized, responsive Web app that delivers content through 3 major components as follows: scenario-based vignettes to present key concepts on clinical trials, informed consent, and other IRB-required material; quiz items to assess the decisional capacity based on the MacCAT-CR; and a tile sorting activity to provide a values clarification exercise at the conclusion of each session. Material for the Decision Aid was developed using input from a multidisciplinary committee of experienced developmental or clinical psychologists, clinicians, and communication scientists, based on data from qualitative interviews conducted with caregivers and individuals with FXS. Technical activities were informed by an agile, user-centered design approach, existing literature on the principles of universal design, and best practices for developing decision aids. The DA was implemented in Hypertext Markup Language 5 using CreateJS libraries for integration of animated multimedia, audio narration, and active tasks. The user interface and user experience were optimized for iOS tablet-based deployment and support interaction through standard touchscreen gestural controls (eg, swipe, tap, drag, and pinch).
A custom case management system enables event logging for each study participant, including case identifier, interaction specifics, and session time stamps. Built-in app analytics report events via JavaScript Object Notation packets sent over Secure Hypertext Transfer Protocol to a data management service that logs events, storing data in an encrypted relational database. Data extraction and reporting are performed by analysts using Structured Query Language queries and scripted transformations to prepare session data for the statistical analysis. FXS Decision Aid sessions lasted approximately 25 minutes and were intended to be deployed under supervised, experimental conditions in a study participant’s home or clinical setting by field staff. Devices used for field deployment were configured and conditioned throughout data collection in a manner consistent with the recommendations for tablet-based digital health intervention research by Furberg et al [43]. A video demonstration of the FXS Decision Aid is available in Multimedia Appendix 1.

5-iii) Revisions and updating

Revisions and updating. Clearly mention the date and/or version number of the application/intervention (and comparator, if applicable) evaluated, or describe whether the intervention underwent major changes during the evaluation process, or whether the development and/or content was "frozen" during the trial. Describe dynamic components such as news feeds or changing content which may have an impact on the replicability of the intervention (for unexpected events see item 3b).

	1	2	3	4	5	
subitem not at all important	(•)	( )	( )	( )	( )	essential

Does your paper address subitem 5-iii?

Copy and paste relevant sections from the manuscript (include quotes in quotation marks "like this" to indicate direct quotes from your manuscript), or elaborate on this item by providing additional information not in the ms, or briefly explain why the item is not applicable/relevant for your study

The intervention material was not updated during the trial.

5-iv) Quality assurance methods

Provide information on quality assurance methods to ensure accuracy and quality of information provided [1], if applicable.

	1	2	3	4	5	
subitem not at all important	( )	( )	(•)	( )	( )	essential

Does your paper address subitem 5-iv?

Copy and paste relevant sections from the manuscript (include quotes in quotation marks "like this" to indicate direct quotes from your manuscript), or elaborate on this item by providing additional information not in the ms, or briefly explain why the item is not applicable/relevant for your study

Design and development of the intervention material and the principles that informed our approach are described elsewhere [42]. The FXS Decision Aid is a tablet-optimized, responsive Web app that delivers content through 3 major components as follows: scenario-based vignettes to present key concepts on clinical trials, informed consent, and other IRB-required material; quiz items to assess the decisional capacity based on the MacCAT-CR; and a tile sorting activity to provide a values clarification exercise at the conclusion of each session. Material for the Decision Aid was developed using input from a multidisciplinary committee of experienced developmental or clinical psychologists, clinicians, and communication scientists, based on data from qualitative interviews conducted with caregivers and individuals with FXS. Technical activities were informed by an agile, user-centered design approach, existing literature on the principles of universal design, and best practices for developing decision aids. The DA was implemented in Hypertext Markup Language 5 using CreateJS libraries for integration of animated multimedia, audio narration, and active tasks. The user interface and user experience were optimized for iOS tablet-based deployment and support interaction through standard touchscreen gestural controls (eg, swipe, tap, drag, and pinch).
A custom case management system enables event logging for each study participant, including case identifier, interaction specifics, and session time stamps. Built-in app analytics report events via JavaScript Object Notation packets sent over Secure Hypertext Transfer Protocol to a data management service that logs events, storing data in an encrypted relational database. Data extraction and reporting are performed by analysts using Structured Query Language queries and scripted transformations to prepare session data for the statistical analysis. FXS Decision Aid sessions lasted approximately 25 minutes and were intended to be deployed under supervised, experimental conditions in a study participant’s home or clinical setting by field staff. Devices used for field deployment were configured and conditioned throughout data collection in a manner consistent with the recommendations for tablet-based digital health intervention research by Furberg et al [43]. A video demonstration of the FXS Decision Aid is available in Multimedia Appendix 1.

5-v) Ensure replicability by publishing the source code, and/or providing screenshots/screen-capture video, and/or providing flowcharts of the algorithms used

Ensure replicability by publishing the source code, and/or providing screenshots/screen-capture video, and/or providing flowcharts of the algorithms used. Replicability (i.e., other researchers should in principle be able to replicate the study) is a hallmark of scientific reporting.

	1	2	3	4	5	
subitem not at all important	○	○	●	○	○	essential

Does your paper address subitem 5-v?

Copy and paste relevant sections from the manuscript (include quotes in quotation marks "like this" to indicate direct quotes from your manuscript), or elaborate on this item by providing additional information not in the ms, or briefly explain why the item is not applicable/relevant for your study

Design and development of the intervention material and the principles that informed our approach are described elsewhere [42]. The FXS Decision Aid is a tablet-optimized, responsive Web app that delivers content through 3 major components as follows: scenario-based vignettes to present key concepts on clinical trials, informed consent, and other IRB-required material; quiz items to assess the decisional capacity based on the MacCAT-CR; and a tile sorting activity to provide a values clarification exercise at the conclusion of each session. Material for the Decision Aid was developed using input from a multidisciplinary committee of experienced developmental or clinical psychologists, clinicians, and communication scientists, based on data from qualitative interviews conducted with caregivers and individuals with FXS. Technical activities were informed by an agile, user-centered design approach, existing literature on the principles of universal design, and best practices for developing decision aids. The DA was implemented in Hypertext Markup Language 5 using CreateJS libraries for integration of animated multimedia, audio narration, and active tasks. The user interface and user experience were optimized for iOS tablet-based deployment and support interaction through standard touchscreen gestural controls (eg, swipe, tap, drag, and pinch).
A custom case management system enables event logging for each study participant, including case identifier, interaction specifics, and session time stamps. Built-in app analytics report events via JavaScript Object Notation packets sent over Secure Hypertext Transfer Protocol to a data management service that logs events, storing data in an encrypted relational database. Data extraction and reporting are performed by analysts using Structured Query Language queries and scripted transformations to prepare session data for the statistical analysis. FXS Decision Aid sessions lasted approximately 25 minutes and were intended to be deployed under supervised, experimental conditions in a study participant’s home or clinical setting by field staff. Devices used for field deployment were configured and conditioned throughout data collection in a manner consistent with the recommendations for tablet-based digital health intervention research by Furberg et al [43]. A video demonstration of the FXS Decision Aid is available in Multimedia Appendix 1.

5-vi) Digital preservation

Digital preservation: Provide the URL of the application, but as the intervention is likely to change or disappear over the course of the years; also make sure the intervention is archived (Internet Archive, webcitation.org, and/or publishing the source code or screenshots/videos alongside the article). As pages behind login screens cannot be archived, consider creating demo pages which are accessible without login.

	1	2	3	4	5	
subitem not at all important	○	○	◉	○	○	essential

Does your paper address subitem 5-vi?

Copy and paste relevant sections from the manuscript (include quotes in quotation marks "like this" to indicate direct quotes from your manuscript), or elaborate on this item by providing additional information not in the ms, or briefly explain why the item is not applicable/relevant for your study

Design and development of the intervention material and the principles that informed our approach are described elsewhere [42]. The FXS Decision Aid is a tablet-optimized, responsive Web app that delivers content through 3 major components as follows: scenario-based vignettes to present key concepts on clinical trials, informed consent, and other IRB-required material; quiz items to assess the decisional capacity based on the MacCAT-CR; and a tile sorting activity to provide a values clarification exercise at the conclusion of each session. Material for the Decision Aid was developed using input from a multidisciplinary committee of experienced developmental or clinical psychologists, clinicians, and communication scientists, based on data from qualitative interviews conducted with caregivers and individuals with FXS. Technical activities were informed by an agile, user-centered design approach, existing literature on the principles of universal design, and best practices for developing decision aids. The DA was implemented in Hypertext Markup Language 5 using CreateJS libraries for integration of animated multimedia, audio narration, and active tasks. The user interface and user experience were optimized for iOS tablet-based deployment and support interaction through standard touchscreen gestural controls (eg, swipe, tap, drag, and pinch).
A custom case management system enables event logging for each study participant, including case identifier, interaction specifics, and session time stamps. Built-in app analytics report events via JavaScript Object Notation packets sent over Secure Hypertext Transfer Protocol to a data management service that logs events, storing data in an encrypted relational database. Data extraction and reporting are performed by analysts using Structured Query Language queries and scripted transformations to prepare session data for the statistical analysis. FXS Decision Aid sessions lasted approximately 25 minutes and were intended to be deployed under supervised, experimental conditions in a study participant’s home or clinical setting by field staff. Devices used for field deployment were configured and conditioned throughout data collection in a manner consistent with the recommendations for tablet-based digital health intervention research by Furberg et al [43]. A video demonstration of the FXS Decision Aid is available in Multimedia Appendix 1.

5-vii) Access

Access: Describe how participants accessed the application, in what setting/context, if they had to pay (or were paid) or not, whether they had to be a member of specific group. If known, describe how participants obtained “access to the platform and Internet” [1]. To ensure access for editors/reviewers/readers, consider to provide a “backdoor” login account or demo mode for reviewers/readers to explore the application (also important for archiving purposes, see vi).

	1	2	3	4	5	
subitem not at all important	○	○	◉	○	○	essential

Does your paper address subitem 5-vii? *

Copy and paste relevant sections from the manuscript (include quotes in quotation marks "like this" to indicate direct quotes from your manuscript), or elaborate on this item by providing additional information not in the ms, or briefly explain why the item is not applicable/relevant for your study

Design and development of the intervention material and the principles that informed our approach are described elsewhere [42]. The FXS Decision Aid is a tablet-optimized, responsive Web app that delivers content through 3 major components as follows: scenario-based vignettes to present key concepts on clinical trials, informed consent, and other IRB-required material; quiz items to assess the decisional capacity based on the MacCAT-CR; and a tile sorting activity to provide a values clarification exercise at the conclusion of each session. Material for the Decision Aid was developed using input from a multidisciplinary committee of experienced developmental or clinical psychologists, clinicians, and communication scientists, based on data from qualitative interviews conducted with caregivers and individuals with FXS. Technical activities were informed by an agile, user-centered design approach, existing literature on the principles of universal design, and best practices for developing decision aids. The DA was implemented in Hypertext Markup Language 5 using CreateJS libraries for integration of animated multimedia, audio narration, and active tasks. The user interface and user experience were optimized for iOS tablet-based deployment and support interaction through standard touchscreen gestural controls (eg, swipe, tap, drag, and pinch).
A custom case management system enables event logging for each study participant, including case identifier, interaction specifics, and session time stamps. Built-in app analytics report events via JavaScript Object Notation packets sent over Secure Hypertext Transfer Protocol to a data management service that logs events, storing data in an encrypted relational database. Data extraction and reporting are performed by analysts using Structured Query Language queries and scripted transformations to prepare session data for the statistical analysis. FXS Decision Aid sessions lasted approximately 25 minutes and were intended to be deployed under supervised, experimental conditions in a study participant’s home or clinical setting by field staff. Devices used for field deployment were configured and conditioned throughout data collection in a manner consistent with the recommendations for tablet-based digital health intervention research by Furberg et al [43]. A video demonstration of the FXS Decision Aid is available in Multimedia Appendix 1.

5-viii) Mode of delivery, features/functionalities/components of the intervention and comparator, and the theoretical framework

Describe mode of delivery, features/functionalities/components of the intervention and comparator, and the theoretical framework [6] used to design them (instructional strategy [1], behaviour change techniques, persuasive features, etc., see e.g., [7, 8] for terminology). This includes an in-depth description of the content (including where it is coming from and who developed it) [1],” whether [and how] it is tailored to individual circumstances and allows users to track their progress and receive feedback” [6]. This also includes a description of communication delivery channels and – if computer-mediated communication is a component – whether communication was synchronous or asynchronous [6]. It also includes information on presentation strategies [1], including page design principles, average amount of text on pages, presence of hyperlinks to other resources, etc. [1].

	1	2	3	4	5	
subitem not at all important	○	○	◉	○	○	essential

Does your paper address subitem 5-viii? *

Copy and paste relevant sections from the manuscript (include quotes in quotation marks "like this" to indicate direct quotes from your manuscript), or elaborate on this item by providing additional information not in the ms, or briefly explain why the item is not applicable/relevant for your study

Design and development of the intervention material and the principles that informed our approach are described elsewhere [42]. The FXS Decision Aid is a tablet-optimized, responsive Web app that delivers content through 3 major components as follows: scenario-based vignettes to present key concepts on clinical trials, informed consent, and other IRB-required material; quiz items to assess the decisional capacity based on the MacCAT-CR; and a tile sorting activity to provide a values clarification exercise at the conclusion of each session. Material for the Decision Aid was developed using input from a multidisciplinary committee of experienced developmental or clinical psychologists, clinicians, and communication scientists, based on data from qualitative interviews conducted with caregivers and individuals with FXS. Technical activities were informed by an agile, user-centered design approach, existing literature on the principles of universal design, and best practices for developing decision aids. The DA was implemented in Hypertext Markup Language 5 using CreateJS libraries for integration of animated multimedia, audio narration, and active tasks. The user interface and user experience were optimized for iOS tablet-based deployment and support interaction through standard touchscreen gestural controls (eg, swipe, tap, drag, and pinch).
A custom case management system enables event logging for each study participant, including case identifier, interaction specifics, and session time stamps. Built-in app analytics report events via JavaScript Object Notation packets sent over Secure Hypertext Transfer Protocol to a data management service that logs events, storing data in an encrypted relational database. Data extraction and reporting are performed by analysts using Structured Query Language queries and scripted transformations to prepare session data for the statistical analysis. FXS Decision Aid sessions lasted approximately 25 minutes and were intended to be deployed under supervised, experimental conditions in a study participant's home or clinical setting by field staff. Devices used for field deployment were configured and conditioned throughout data collection in a manner consistent with the recommendations for tablet-based digital health intervention research by Furberg et al [43]. A video demonstration of the FXS Decision Aid is available in Multimedia Appendix 1.

5-ix) Describe use parameters

Describe use parameters (e.g., intended “doses” and optimal timing for use). Clarify what instructions or recommendations were given to the user, e.g., regarding timing, frequency, heaviness of use, if any, or was the intervention used ad libitum.

	1	2	3	4	5	
subitem not at all important	○	○	◉	○	○	essential

Does your paper address subitem 5-ix?

Copy and paste relevant sections from the manuscript (include quotes in quotation marks "like this" to indicate direct quotes from your manuscript), or elaborate on this item by providing additional information not in the ms, or briefly explain why the item is not applicable/relevant for your study

Design and development of the intervention material and the principles that informed our approach are described elsewhere [42]. The FXS Decision Aid is a tablet-optimized, responsive Web app that delivers content through 3 major components as follows: scenario-based vignettes to present key concepts on clinical trials, informed consent, and other IRB-required material; quiz items to assess the decisional capacity based on the MacCAT-CR; and a tile sorting activity to provide a values clarification exercise at the conclusion of each session. Material for the Decision Aid was developed using input from a multidisciplinary committee of experienced developmental or clinical psychologists, clinicians, and communication scientists, based on data from qualitative interviews conducted with caregivers and individuals with FXS. Technical activities were informed by an agile, user-centered design approach, existing literature on the principles of universal design, and best practices for developing decision aids. The DA was implemented in Hypertext Markup Language 5 using CreateJS libraries for integration of animated multimedia, audio narration, and active tasks. The user interface and user experience were optimized for iOS tablet-based deployment and support interaction through standard touchscreen gestural controls (eg, swipe, tap, drag, and pinch).
A custom case management system enables event logging for each study participant, including case identifier, interaction specifics, and session time stamps. Built-in app analytics report events via JavaScript Object Notation packets sent over Secure Hypertext Transfer Protocol to a data management service that logs events, storing data in an encrypted relational database. Data extraction and reporting are performed by analysts using Structured Query Language queries and scripted transformations to prepare session data for the statistical analysis. FXS Decision Aid sessions lasted approximately 25 minutes and were intended to be deployed under supervised, experimental conditions in a study participant's home or clinical setting by field staff. Devices used for field deployment were configured and conditioned throughout data collection in a manner consistent with the recommendations for tablet-based digital health intervention research by Furberg et al [43]. A video demonstration of the FXS Decision Aid is available in Multimedia Appendix 1.

5-x) Clarify the level of human involvement

Clarify the level of human involvement (care providers or health professionals, also technical assistance) in the e-intervention or as co-intervention (detail number and expertise of professionals involved, if any, as well as "type of assistance offered, the timing and frequency of the support, how it is initiated, and the medium by which the assistance is delivered". It may be necessary to distinguish between the level of human involvement required for the trial, and the level of human involvement required for a routine application outside of a RCT setting (discuss under item 21 – generalizability).

	1	2	3	4	5	
subitem not at all important	○	○	◉	○	○	essential

Does your paper address subitem 5-x?

Copy and paste relevant sections from the manuscript (include quotes in quotation marks "like this" to indicate direct quotes from your manuscript), or elaborate on this item by providing additional information not in the ms, or briefly explain why the item is not applicable/relevant for your study

Design and development of the intervention material and the principles that informed our approach are described elsewhere [42]. The FXS Decision Aid is a tablet-optimized, responsive Web app that delivers content through 3 major components as follows: scenario-based vignettes to present key concepts on clinical trials, informed consent, and other IRB-required material; quiz items to assess the decisional capacity based on the MacCAT-CR; and a tile sorting activity to provide a values clarification exercise at the conclusion of each session. Material for the Decision Aid was developed using input from a multidisciplinary committee of experienced developmental or clinical psychologists, clinicians, and communication scientists, based on data from qualitative interviews conducted with caregivers and individuals with FXS. Technical activities were informed by an agile, user-centered design approach, existing literature on the principles of universal design, and best practices for developing decision aids. The DA was implemented in Hypertext Markup Language 5 using CreateJS libraries for integration of animated multimedia, audio narration, and active tasks. The user interface and user experience were optimized for iOS tablet-based deployment and support interaction through standard touchscreen gestural controls (eg, swipe, tap, drag, and pinch).
A custom case management system enables event logging for each study participant, including case identifier, interaction specifics, and session time stamps. Built-in app analytics report events via JavaScript Object Notation packets sent over Secure Hypertext Transfer Protocol to a data management service that logs events, storing data in an encrypted relational database. Data extraction and reporting are performed by analysts using Structured Query Language queries and scripted transformations to prepare session data for the statistical analysis. FXS Decision Aid sessions lasted approximately 25 minutes and were intended to be deployed under supervised, experimental conditions in a study participant's home or clinical setting by field staff. Devices used for field deployment were configured and conditioned throughout data collection in a manner consistent with the recommendations for tablet-based digital health intervention research by Furberg et al [43]. A video demonstration of the FXS Decision Aid is available in Multimedia Appendix 1.

5-xi) Report any prompts/reminders used

Report any prompts/reminders used: Clarify if there were prompts (letters, emails, phone calls, SMS) to use the application, what triggered them, frequency etc. It may be necessary to distinguish between the level of prompts/reminders required for the trial, and the level of prompts/reminders for a routine application outside of a RCT setting (discuss under item 21 – generalizability).

	1	2	3	4	5	
subitem not at all important	(•)	( )	( )	( )	( )	essential

Does your paper address subitem 5-xi? *

Copy and paste relevant sections from the manuscript (include quotes in quotation marks "like this" to indicate direct quotes from your manuscript), or elaborate on this item by providing additional information not in the ms, or briefly explain why the item is not applicable/relevant for your study

Not applicable

5-xii) Describe any co-interventions (incl. training/support)

Describe any co-interventions (incl. training/support): Clearly state any interventions that are provided in addition to the targeted eHealth intervention, as ehealth intervention may not be designed as stand-alone intervention. This includes training sessions and support [1]. It may be necessary to distinguish between the level of training required for the trial, and the level of training for a routine application outside of a RCT setting (discuss under item 21 – generalizability.

	1	2	3	4	5	
subitem not at all important	( )	( )	( )	( )	( )	essential

Does your paper address subitem 5-xii? *

Copy and paste relevant sections from the manuscript (include quotes in quotation marks "like this" to indicate direct quotes from your manuscript), or elaborate on this item by providing additional information not in the ms, or briefly explain why the item is not applicable/relevant for your study

"Each study session will occur in the individual’s home and will be videotaped to allow subsequent coding of individual engagement in the decisional process. Approximately 10 days prior to the visit, the participant or their primary caregiver will be sent the standard paper consent form for a hypothetical clinical trial. All participants, including parents and caregivers, will be informed that the clinical trial is hypothetical. Participants will be asked to review the consent form as they would any research consent form.
On the day of the visit, parents and individuals with FXS who are their own legal guardian will be asked to complete a 5- to 10-minute pretest to assess their or their child’s belief and attitudes about participating in clinical trials. Pretest items included questions such as participants’ possible reasons for participating in clinical trials, the likelihood of participating, and whom they think should make the decision about enrolling in a trial (full list of domains in “Study Outcomes” section below). Participants will then complete either the experimental or control group informed consent procedure. Both the control and experimental groups will be asked the modified MacCAT examination items throughout the informed consent process to assess the decisional capacity. The questions are identical for each group. After the informed consent procedure, all participants will again be asked to complete the posttest questions about their beliefs and attitudes about clinical trials."

6a) Completely defined pre-specified primary and secondary outcome measures, including how and when they were assessed

Does your paper address CONSORT subitem 6a? *

Copy and paste relevant sections from the manuscript (include quotes in quotation marks "like this" to indicate direct quotes from your manuscript), or elaborate on this item by providing additional information not in the ms, or briefly explain why the item is not applicable/relevant for your study

"The following measures will be collected as part of the study protocol: time spent with decision aid or in discussion with research assistant; perceptions about reasons to participate in trials (eg, altruism; pre- and posttest item); likelihood of enrolling in a clinical trial (pre- and posttest item); the preferred level of involvement in the decision (pre- and posttest item); self-efficacy related to decision-making ability (pre- and posttest item); level of engagement in the decision-making process (pre- and posttest item);
satisfaction with the decision (posttest only); the perceived value of the educational information provided (posttest only); and session analytics, including time on page and session duration."

6a-i) Online questionnaires: describe if they were validated for online use and apply CHERRIES items to describe how the questionnaires were designed/deployed

If outcomes were obtained through online questionnaires, describe if they were validated for online use and apply CHERRIES items to describe how the questionnaires were designed/deployed [9].

	1	2	3	4	5	
subitem not at all important	(●)	( )	( )	( )	( )	essential

Does your paper address subitem 6a-i?

Copy and paste relevant sections from manuscript text

N/A

6a-ii) Describe whether and how “use” (including intensity of use/dosage) was defined/measured/monitored

Describe whether and how “use” (including intensity of use/dosage) was defined/measured/monitored (logins, logfile analysis, etc.). Use/adoption metrics are important process outcomes that should be reported in any ehealth trial.

	1	2	3	4	5	
subitem not at all important	○	○	●	○	○	essential

Does your paper address subitem 6a-ii?

Copy and paste relevant sections from manuscript text

"Pretest measures, the intervention or control condition, and posttest measures will all be conducted on the same day during an approximately 1-hour study session. The pre- and posttest measures will take approximately 15 minutes to complete, and the control and intervention condition will last approximately 30 minutes. No additional follow-up is planned."

6a-iii) Describe whether, how, and when qualitative feedback from participants was obtained

Describe whether, how, and when qualitative feedback from participants was obtained (e.g., through emails, feedback forms, interviews, focus groups).

	1	2	3	4	5	
subitem not at all important	●	○	○	○	○	essential

Does your paper address subitem 6a-iii?

Copy and paste relevant sections from manuscript text

N/A

6b) Any changes to trial outcomes after the trial commenced, with reasons

Does your paper address CONSORT subitem 6b? *

Copy and paste relevant sections from the manuscript (include quotes in quotation marks "like this" to indicate direct quotes from your manuscript), or elaborate on this item by providing additional information not in the ms, or briefly explain why the item is not applicable/relevant for your study

Not applicable

7a) How sample size was determined

NPT: When applicable, details of whether and how the clustering by care provides or centers was addressed

7a-i) Describe whether and how expected attrition was taken into account when calculating the sample size

Describe whether and how expected attrition was taken into account when calculating the sample size.

	1	2	3	4	5	
subitem not at all important	○	○	◉	○	○	essential

Does your paper address subitem 7a-i?

Copy and paste relevant sections from manuscript title (include quotes in quotation marks "like this" to indicate direct quotes from your manuscript), or elaborate on this item by providing additional information not in the ms, or briefly explain why the item is not applicable/relevant for your study

"We have conducted a power analysis using a between-subjects design (ie, participants are randomized to the control or experimental group) to determine the recommended sample size. With a sample size of 70 (35 participants per group), we will have 90% power to detect an effect size of 0.75."

7b) When applicable, explanation of any interim analyses and stopping guidelines

Does your paper address CONSORT subitem 7b? *

Copy and paste relevant sections from the manuscript (include quotes in quotation marks "like this" to indicate direct quotes from your manuscript), or elaborate on this item by providing additional information not in the ms, or briefly explain why the item is not applicable/relevant for your study

Not applicable

8a) Method used to generate the random allocation sequence

NPT: When applicable, how care providers were allocated to each trial group

Does your paper address CONSORT subitem 8a? *

Copy and paste relevant sections from the manuscript (include quotes in quotation marks "like this" to indicate direct quotes from your manuscript), or elaborate on this item by providing additional information not in the ms, or briefly explain why the item is not applicable/relevant for your study

Your answer

8b) Type of randomisation; details of any restriction (such as blocking and block size)

Does your paper address CONSORT subitem 8b? *

Copy and paste relevant sections from the manuscript (include quotes in quotation marks "like this" to indicate direct quotes from your manuscript), or elaborate on this item by providing additional information not in the ms, or briefly explain why the item is not applicable/relevant for your study

"We will use a stratified, block randomization method to assign participants to the control or experimental group. Two stratification variables will be used—verbal IQ score (3 levels) and age (2 levels). Age was selected as a randomizing variable because children and adolescents under 18 are not able to provide informed consent, only assent, as their parents are their legal guardians. Verbal IQ was selected as a second variable because we had a small sample size and wanted to control for any possible effects on the outcome variables. However, we could have also chosen to account for any possible group differences based on IQ through statistical analyses. Given that enrollment for the RCT will be done on a rolling basis, a 10-block, 2-group design will be used. Thus, we will randomize 10 participants at a time into either the control or experimental group. Furthermore, we will utilize a random number generator (www.randomizer.com) to make the assignments."

9) Mechanism used to implement the random allocation sequence (such as sequentially numbered containers), describing any steps taken to conceal the sequence until interventions were assigned

Does your paper address CONSORT subitem 9? *

Copy and paste relevant sections from the manuscript (include quotes in quotation marks "like this" to indicate direct quotes from your manuscript), or elaborate on this item by providing additional information not in the ms, or briefly explain why the item is not applicable/relevant for your study

"We will use a stratified, block randomization method to assign participants to the control or experimental group. Two stratification variables will be used—verbal IQ score (3 levels) and age (2 levels). Age was selected as a randomizing variable because children and adolescents under 18 are not able to provide informed consent, only assent, as their parents are their legal guardians. Verbal IQ was selected as a second variable because we had a small sample size and wanted to control for any possible effects on the outcome variables. However, we could have also chosen to account for any possible group differences based on IQ through statistical analyses. Given that enrollment for the RCT will be done on a rolling basis, a 10-block, 2-group design will be used. Thus, we will randomize 10 participants at a time into either the control or experimental group. Furthermore, we will utilize a random number generator (www.randomizer.com) to make the assignments."

10) Who generated the random allocation sequence, who enrolled participants, and who assigned participants to interventions

Does your paper address CONSORT subitem 10? *

Copy and paste relevant sections from the manuscript (include quotes in quotation marks "like this" to indicate direct quotes from your manuscript), or elaborate on this item by providing additional information not in the ms, or briefly explain why the item is not applicable/relevant for your study

Authors

11a) If done, who was blinded after assignment to interventions (for example, participants, care providers, those assessing outcomes) and how

NPT: Whether or not administering co-interventions were blinded to group assignment

11a-i) Specify who was blinded, and who wasn’t

Specify who was blinded, and who wasn’t. Usually, in web-based trials it is not possible to blind the participants [1, 3] (this should be clearly acknowledged), but it may be possible to blind outcome assessors, those doing data analysis or those administering co-interventions (if any).

	1	2	3	4	5	
subitem not at all important	○	○	◉	○	○	essential

Does your paper address subitem 11a-i? *

Copy and paste relevant sections from the manuscript (include quotes in quotation marks "like this" to indicate direct quotes from your manuscript), or elaborate on this item by providing additional information not in the ms, or briefly explain why the item is not applicable/relevant for your study

"Owing to the nature of the study, participants and data collectors were not blinded to the group assignment."

11a-ii) Discuss e.g., whether participants knew which intervention was the “intervention of interest” and which one was the “comparator”

Informed consent procedures (4a-ii) can create biases and certain expectations - discuss e.g., whether participants knew which intervention was the “intervention of interest” and which one was the “comparator”.

	1	2	3	4	5	
subitem not at all important	○	○	◉	○	○	essential

Does your paper address subitem 11a-ii?

Copy and paste relevant sections from the manuscript (include quotes in quotation marks "like this" to indicate direct quotes from your manuscript), or elaborate on this item by providing additional information not in the ms, or briefly explain why the item is not applicable/relevant for your study

"Participants randomized to the experimental group will receive the paper consent form (the same form the control group participants received) and will also be exposed to the tablet-based app. Given that the digital app does not meet the requirements of electronic consent, the paper consent form will still be needed for documenting that informed consent was obtained."

11b) If relevant, description of the similarity of interventions

(this item is usually not relevant for ehealth trials as it refers to similarity of a placebo or sham intervention to a active medication/intervention)

Does your paper address CONSORT subitem 11b? *

Copy and paste relevant sections from the manuscript (include quotes in quotation marks "like this" to indicate direct quotes from your manuscript), or elaborate on this item by providing additional information not in the ms, or briefly explain why the item is not applicable/relevant for your study

"Participants randomized to the control group will be exposed to a paper consent form that covers the same informed consent information presented in the tablet-based app and mimics informed consent forms used for real clinical trials. The complex language that is typically used in current standard practice (ie, participation vs take part) will be retained in the paper version. The control group will be designed to mimic typical informed consent procedures, that is, the verbal transmission of study information from a clinician to the individual and caregivers. To standardize this practice, we will develop a script that is verbally reviewed with control group participants, including a simplified overview of the key information in the paper consent form."

12a) Statistical methods used to compare groups for primary and secondary outcomes

NPT: When applicable, details of whether and how the clustering by care providers or centers was addressed

Does your paper address CONSORT subitem 12a? *

Copy and paste relevant sections from the manuscript (include quotes in quotation marks "like this" to indicate direct quotes from your manuscript), or elaborate on this item by providing additional information not in the ms, or briefly explain why the item is not applicable/relevant for your study

"In the analyses, we will examine the effect of the app on the decisional capacity, controlling for sociodemographic characteristics and severity of delay. We will first conduct bivariate analyses comparing the decisional capacity, decision-making preferences, and the likelihood of trial participation across the 2 study groups (tablet vs standard procedure), using chi-square tests for categorical outcomes and t tests for continuous outcomes. In addition, we will conduct multiple regression models to compare study outcomes by the study group after controlling for demographics and severity of developmental delay. Linear regression models will be conducted for continuous outcomes (eg, decisional capacity scores and preferences) and logistic regression models for categorical outcomes (eg, the likelihood of participating in a hypothetical trial). Within these models, we will test for interactions between study group and demographic characteristics to identify the differential impact of the intervention on particular subgroups. For example, testing an interaction between study group and severity of delay would allow us to determine whether the tablet-based intervention is more or less successful among more impaired participants."

12a-i) Imputation techniques to deal with attrition / missing values

Imputation techniques to deal with attrition / missing values: Not all participants will use the intervention/comparator as intended and attrition is typically high in ehealth trials. Specify how participants who did not use the application or dropped out from the trial were treated in the statistical analysis (a complete case analysis is strongly discouraged, and simple imputation techniques such as LOCF may also be problematic [4]).

	1	2	3	4	5	
subitem not at all important	○	○	●	○	○	essential

Does your paper address subitem 12a-i? *

Copy and paste relevant sections from the manuscript (include quotes in quotation marks "like this" to indicate direct quotes from your manuscript), or elaborate on this item by providing additional information not in the ms, or briefly explain why the item is not applicable/relevant for your study

"In the analyses, we will examine the effect of the app on the decisional capacity, controlling for sociodemographic characteristics and severity of delay. We will first conduct bivariate analyses comparing the decisional capacity, decision-making preferences, and the likelihood of trial participation across the 2 study groups (tablet vs standard procedure), using chi-square tests for categorical outcomes and t tests for continuous outcomes. In addition, we will conduct multiple regression models to compare study outcomes by the study group after controlling for demographics and severity of developmental delay. Linear regression models will be conducted for continuous outcomes (eg, decisional capacity scores and preferences) and logistic regression models for categorical outcomes (eg, the likelihood of participating in a hypothetical trial). Within these models, we will test for interactions between study group and demographic characteristics to identify the differential impact of the intervention on particular subgroups. For example, testing an interaction between study group and severity of delay would allow us to determine whether the tablet-based intervention is more or less successful among more impaired participants."

12b) Methods for additional analyses, such as subgroup analyses and adjusted analyses

Does your paper address CONSORT subitem 12b? *

Copy and paste relevant sections from the manuscript (include quotes in quotation marks "like this" to indicate direct quotes from your manuscript), or elaborate on this item by providing additional information not in the ms, or briefly explain why the item is not applicable/relevant for your study

"In the analyses, we will examine the effect of the app on the decisional capacity, controlling for sociodemographic characteristics and severity of delay. We will first conduct bivariate analyses comparing the decisional capacity, decision-making preferences, and the likelihood of trial participation across the 2 study groups (tablet vs standard procedure), using chi-square tests for categorical outcomes and t tests for continuous outcomes. In addition, we will conduct multiple regression models to compare study outcomes by the study group after controlling for demographics and severity of developmental delay. Linear regression models will be conducted for continuous outcomes (eg, decisional capacity scores and preferences) and logistic regression models for categorical outcomes (eg, the likelihood of participating in a hypothetical trial). Within these models, we will test for interactions between study group and demographic characteristics to identify the differential impact of the intervention on particular subgroups. For example, testing an interaction between study group and severity of delay would allow us to determine whether the tablet-based intervention is more or less successful among more impaired participants."

X26) REB/IRB Approval and Ethical Considerations [recommended as subheading under "Methods"] (not a CONSORT item)

X26-i) Comment on ethics committee approval

	1	2	3	4	5	
subitem not at all important	○	○	◉	○	○	essential

Does your paper address subitem X26-i?

Copy and paste relevant sections from the manuscript (include quotes in quotation marks "like this" to indicate direct quotes from your manuscript), or elaborate on this item by providing additional information not in the ms, or briefly explain why the item is not applicable/relevant for your study

"This research protocol was reviewed and approved by 2 Independent Review Boards from the University of North Carolina Office of Human Research Ethics (IRB Number: 13-1128) and RTI International Office of Research Protection (0281200.276). This study has been registered with ClinicalTrials.gov (NCT02465931)."

x26-ii) Outline informed consent procedures

Outline informed consent procedures e.g., if consent was obtained offline or online (how? Checkbox, etc.?), and what information was provided (see 4a-ii). See [6] for some items to be included in informed consent documents.

	1	2	3	4	5	
subitem not at all important	○	○	◉	○	○	essential

Does your paper address subitem X26-ii?

Copy and paste relevant sections from the manuscript (include quotes in quotation marks "like this" to indicate direct quotes from your manuscript), or elaborate on this item by providing additional information not in the ms, or briefly explain why the item is not applicable/relevant for your study

"Each study session will occur in the individual's home and will be videotaped to allow subsequent coding of individual engagement in the decisional process. Approximately 10 days prior to the visit, the participant or their primary caregiver will be sent the standard paper consent form for a hypothetical clinical trial. All participants, including parents and caregivers, will be informed that the clinical trial is hypothetical. Participants will be asked to review the consent form as they would any research consent form."

X26-iii) Safety and security procedures

Safety and security procedures, incl. privacy considerations, and any steps taken to reduce the likelihood or detection of harm (e.g., education and training, availability of a hotline)

	1	2	3	4	5	
subitem not at all important	○	○	○	○	○	essential

Does your paper address subitem X26-iii?

Copy and paste relevant sections from the manuscript (include quotes in quotation marks "like this" to indicate direct quotes from your manuscript), or elaborate on this item by providing additional information not in the ms, or briefly explain why the item is not applicable/relevant for your study

Not applicable

RESULTS

13a) For each group, the numbers of participants who were randomly assigned, received intended treatment, and were analysed for the primary outcome

NPT: The number of care providers or centers performing the intervention in each group and the number of patients treated by each care provider in each center

Does your paper address CONSORT subitem 13a? *

Copy and paste relevant sections from the manuscript (include quotes in quotation marks "like this" to indicate direct quotes from your manuscript), or elaborate on this item by providing additional information not in the ms, or briefly explain why the item is not applicable/relevant for your study

Not applicable

13b) For each group, losses and exclusions after randomisation, together with reasons

Does your paper address CONSORT subitem 13b? (NOTE: Preferably, this is shown in a CONSORT flow diagram) *

Copy and paste relevant sections from the manuscript (include quotes in quotation marks "like this" to indicate direct quotes from your manuscript), or elaborate on this item by providing additional information not in the ms, or briefly explain why the item is not applicable/relevant for your study

Not applicable

13b-i) Attrition diagram

Strongly recommended: An attrition diagram (e.g., proportion of participants still logging in or using the intervention/comparator in each group plotted over time, similar to a survival curve) or other figures or tables demonstrating usage/dose/engagement.

	1	2	3	4	5	
subitem not at all important	○	○	◉	○	○	essential

Does your paper address subitem 13b-i?

Copy and paste relevant sections from the manuscript or cite the figure number if applicable (include quotes in quotation marks "like this" to indicate direct quotes from your manuscript), or elaborate on this item by providing additional information not in the ms, or briefly explain why the item is not applicable/relevant for your study

Not applicable

14a) Dates defining the periods of recruitment and follow-up

Does your paper address CONSORT subitem 14a? *

Copy and paste relevant sections from the manuscript (include quotes in quotation marks "like this" to indicate direct quotes from your manuscript), or elaborate on this item by providing additional information not in the ms, or briefly explain why the item is not applicable/relevant for your study

Not applicable

14a-i) Indicate if critical “secular events” fell into the study period

Indicate if critical “secular events” fell into the study period, e.g., significant changes in Internet resources available or “changes in computer hardware or Internet delivery resources”

	1	2	3	4	5	
subitem not at all important	○	○	◉	○	○	essential

Does your paper address subitem 14a-i?

Copy and paste relevant sections from the manuscript (include quotes in quotation marks "like this" to indicate direct quotes from your manuscript), or elaborate on this item by providing additional information not in the ms, or briefly explain why the item is not applicable/relevant for your study

Not applicable

14b) Why the trial ended or was stopped (early)

Does your paper address CONSORT subitem 14b? *

Copy and paste relevant sections from the manuscript (include quotes in quotation marks "like this" to indicate direct quotes from your manuscript), or elaborate on this item by providing additional information not in the ms, or briefly explain why the item is not applicable/relevant for your study

Not applicable

15) A table showing baseline demographic and clinical characteristics for each group

NPT: When applicable, a description of care providers (case volume, qualification, expertise, etc.) and centers (volume) in each group

Does your paper address CONSORT subitem 15? *

Copy and paste relevant sections from the manuscript (include quotes in quotation marks "like this" to indicate direct quotes from your manuscript), or elaborate on this item by providing additional information not in the ms, or briefly explain why the item is not applicable/relevant for your study

Not applicable

15-i) Report demographics associated with digital divide issues

In ehealth trials it is particularly important to report demographics associated with digital divide issues, such as age, education, gender, social-economic status, computer/Internet/ehealth literacy of the participants, if known.

	1	2	3	4	5	
subitem not at all important	○	○	●	○	○	essential

Does your paper address subitem 15-i? *

Copy and paste relevant sections from the manuscript (include quotes in quotation marks "like this" to indicate direct quotes from your manuscript), or elaborate on this item by providing additional information not in the ms, or briefly explain why the item is not applicable/relevant for your study

Not applicable

16) For each group, number of participants (denominator) included in each analysis and whether the analysis was by original assigned groups

16-i) Report multiple “denominators” and provide definitions

Report multiple “denominators” and provide definitions: Report N’s (and effect sizes) “across a range of study participation [and use] thresholds” [1], e.g., N exposed, N consented, N used more than x times, N used more than y weeks, N participants “used” the intervention/comparator at specific pre-defined time points of interest (in absolute and relative numbers per group). Always clearly define “use” of the intervention.

	1	2	3	4	5	
subitem not at all important	○	○	◉	○	○	essential

Does your paper address subitem 16-i? *

Copy and paste relevant sections from the manuscript (include quotes in quotation marks "like this" to indicate direct quotes from your manuscript), or elaborate on this item by providing additional information not in the ms, or briefly explain why the item is not applicable/relevant for your study

Not applicable

16-ii) Primary analysis should be intent-to-treat

Primary analysis should be intent-to-treat, secondary analyses could include comparing only “users”, with the appropriate caveats that this is no longer a randomized sample (see 18-i).

	1	2	3	4	5	
subitem not at all important	○	○	◉	○	○	essential

Does your paper address subitem 16-ii?

Copy and paste relevant sections from the manuscript (include quotes in quotation marks "like this" to indicate direct quotes from your manuscript), or elaborate on this item by providing additional information not in the ms, or briefly explain why the item is not applicable/relevant for your study

Not applicable

17a) For each primary and secondary outcome, results for each group, and the estimated effect size and its precision (such as 95% confidence interval)

Does your paper address CONSORT subitem 17a? *

Copy and paste relevant sections from the manuscript (include quotes in quotation marks "like this" to indicate direct quotes from your manuscript), or elaborate on this item by providing additional information not in the ms, or briefly explain why the item is not applicable/relevant for your study

Not applicable

17a-i) Presentation of process outcomes such as metrics of use and intensity of use

In addition to primary/secondary (clinical) outcomes, the presentation of process outcomes such as metrics of use and intensity of use (dose, exposure) and their operational definitions is critical. This does not only refer to metrics of attrition (13-b) (often a binary variable), but also to more continuous exposure metrics such as “average session length”. These must be accompanied by a technical description how a metric like a “session” is defined (e.g., timeout after idle time) [1] (report under item 6a).

	1	2	3	4	5	
subitem not at all important	○	○	◉	○	○	essential

Does your paper address subitem 17a-i?

Copy and paste relevant sections from the manuscript (include quotes in quotation marks "like this" to indicate direct quotes from your manuscript), or elaborate on this item by providing additional information not in the ms, or briefly explain why the item is not applicable/relevant for your study

Not applicable

17b) For binary outcomes, presentation of both absolute and relative effect sizes is recommended

Does your paper address CONSORT subitem 17b? *

Copy and paste relevant sections from the manuscript (include quotes in quotation marks "like this" to indicate direct quotes from your manuscript), or elaborate on this item by providing additional information not in the ms, or briefly explain why the item is not applicable/relevant for your study

Not applicable

18) Results of any other analyses performed, including subgroup analyses and adjusted analyses, distinguishing pre-specified from exploratory

Does your paper address CONSORT subitem 18? *

Copy and paste relevant sections from the manuscript (include quotes in quotation marks "like this" to indicate direct quotes from your manuscript), or elaborate on this item by providing additional information not in the ms, or briefly explain why the item is not applicable/relevant for your study

Not applicable

18-i) Subgroup analysis of comparing only users

A subgroup analysis of comparing only users is not uncommon in ehealth trials, but if done, it must be stressed that this is a self-selected sample and no longer an unbiased sample from a randomized trial (see 16-iii).

	1	2	3	4	5	
subitem not at all important	○	○	◉	○	○	essential

Does your paper address subitem 18-i?

Copy and paste relevant sections from the manuscript (include quotes in quotation marks "like this" to indicate direct quotes from your manuscript), or elaborate on this item by providing additional information not in the ms, or briefly explain why the item is not applicable/relevant for your study

Not applicable

19) All important harms or unintended effects in each group

(for specific guidance see CONSORT for harms)

Does your paper address CONSORT subitem 19? *

Copy and paste relevant sections from the manuscript (include quotes in quotation marks "like this" to indicate direct quotes from your manuscript), or elaborate on this item by providing additional information not in the ms, or briefly explain why the item is not applicable/relevant for your study

Not applicable

19-i) Include privacy breaches, technical problems

Include privacy breaches, technical problems. This does not only include physical “harm” to participants, but also incidents such as perceived or real privacy breaches [1], technical problems, and other unexpected/unintended incidents. “Unintended effects” also includes unintended positive effects [2].

	1	2	3	4	5	
subitem not at all important	○	○	●	○	○	essential

Does your paper address subitem 19-i?

Copy and paste relevant sections from the manuscript (include quotes in quotation marks "like this" to indicate direct quotes from your manuscript), or elaborate on this item by providing additional information not in the ms, or briefly explain why the item is not applicable/relevant for your study

Not applicable

19-ii) Include qualitative feedback from participants or observations from staff/researchers

Include qualitative feedback from participants or observations from staff/researchers, if available, on strengths and shortcomings of the application, especially if they point to unintended/unexpected effects or uses. This includes (if available) reasons for why people did or did not use the application as intended by the developers.

	1	2	3	4	5	
subitem not at all important	○	○	●	○	○	essential

Does your paper address subitem 19-ii?

Copy and paste relevant sections from the manuscript (include quotes in quotation marks "like this" to indicate direct quotes from your manuscript), or elaborate on this item by providing additional information not in the ms, or briefly explain why the item is not applicable/relevant for your study

Not applicable

DISCUSSION

22) Interpretation consistent with results, balancing benefits and harms, and considering other relevant evidence

NPT: In addition, take into account the choice of the comparator, lack of or partial blinding, and unequal expertise of care providers or centers in each group

22-i) Restate study questions and summarize the answers suggested by the data, starting with primary outcomes and process outcomes (use)

Restate study questions and summarize the answers suggested by the data, starting with primary outcomes and process outcomes (use).

	1	2	3	4	5	
subitem not at all important	○	○	◉	○	○	essential

Does your paper address subitem 22-i? *

Copy and paste relevant sections from the manuscript (include quotes in quotation marks "like this" to indicate direct quotes from your manuscript), or elaborate on this item by providing additional information not in the ms, or briefly explain why the item is not applicable/relevant for your study

Not applicable

22-ii) Highlight unanswered new questions, suggest future research

Highlight unanswered new questions, suggest future research.

	1	2	3	4	5	
subitem not at all important	○	○	◉	○	○	essential

Does your paper address subitem 22-ii?

Copy and paste relevant sections from the manuscript (include quotes in quotation marks "like this" to indicate direct quotes from your manuscript), or elaborate on this item by providing additional information not in the ms, or briefly explain why the item is not applicable/relevant for your study

Not applicable

20) Trial limitations, addressing sources of potential bias, imprecision, and, if relevant, multiplicity of analyses

20-i) Typical limitations in ehealth trials

Typical limitations in ehealth trials: Participants in ehealth trials are rarely blinded. Ehealth trials often look at a multiplicity of outcomes, increasing risk for a Type I error. Discuss biases due to non-use of the intervention/usability issues, biases through informed consent procedures, unexpected events.

	1	2	3	4	5	
subitem not at all important	○	○	◉	○	○	essential

Does your paper address subitem 20-i? *

Copy and paste relevant sections from the manuscript (include quotes in quotation marks "like this" to indicate direct quotes from your manuscript), or elaborate on this item by providing additional information not in the ms, or briefly explain why the item is not applicable/relevant for your study

Despite the strength of the evaluation design and scalability, the major limitation of this study is the focus on assessing decision making to participate in a hypothetical clinical trial.

21) Generalisability (external validity, applicability) of the trial findings

NPT: External validity of the trial findings according to the intervention, comparators, patients, and care providers or centers involved in the trial

21-i) Generalizability to other populations

Generalizability to other populations: In particular, discuss generalizability to a general Internet population, outside of a RCT setting, and general patient population, including applicability of the study results for other organizations

	1	2	3	4	5	
subitem not at all important	○	○	◉	○	○	essential

Does your paper address subitem 21-i?

Copy and paste relevant sections from the manuscript (include quotes in quotation marks "like this" to indicate direct quotes from your manuscript), or elaborate on this item by providing additional information not in the ms, or briefly explain why the item is not applicable/relevant for your study

Not applicable

21-ii) Discuss if there were elements in the RCT that would be different in a routine application setting

Discuss if there were elements in the RCT that would be different in a routine application setting (e.g., prompts/reminders, more human involvement, training sessions or other co-interventions) and what impact the omission of these elements could have on use, adoption, or outcomes if the intervention is applied outside of a RCT setting.

	1	2	3	4	5	
subitem not at all important	○	○	◉	○	○	essential

Does your paper address subitem 21-ii?

Copy and paste relevant sections from the manuscript (include quotes in quotation marks "like this" to indicate direct quotes from your manuscript), or elaborate on this item by providing additional information not in the ms, or briefly explain why the item is not applicable/relevant for your study

Not applicable

OTHER INFORMATION

23) Registration number and name of trial registry

Does your paper address CONSORT subitem 23? *

Copy and paste relevant sections from the manuscript (include quotes in quotation marks "like this" to indicate direct quotes from your manuscript), or elaborate on this item by providing additional information not in the ms, or briefly explain why the item is not applicable/relevant for your study

This study has been registered with ClinicalTrials.gov (NCT02465931).

24) Where the full trial protocol can be accessed, if available

Does your paper address CONSORT subitem 24? *

Cite a Multimedia Appendix, other reference, or copy and paste relevant sections from the manuscript (include quotes in quotation marks "like this" to indicate direct quotes from your manuscript), or elaborate on this item by providing additional information not in the ms, or briefly explain why the item is not applicable/relevant for your study

Yes

25) Sources of funding and other support (such as supply of drugs), role of funders

Does your paper address CONSORT subitem 25? *

Copy and paste relevant sections from the manuscript (include quotes in quotation marks "like this" to indicate direct quotes from your manuscript), or elaborate on this item by providing additional information not in the ms, or briefly explain why the item is not applicable/relevant for your study

The authors thank the individuals with FXS and their families who participated in this research study. This study was funded by a grant from the Eunice Kennedy Shriver National Institute for Child Health and Human Development (R01HD071987-01A1). The findings and conclusions in this report are those of the authors and do not necessarily represent the official position of the National Institutes of Health.

X27) Conflicts of Interest (not a CONSORT item)

X27-i) State the relation of the study team towards the system being evaluated

In addition to the usual declaration of interests (financial or otherwise), also state the relation of the study team towards the system being evaluated, i.e., state if the authors/evaluators are distinct from or identical with the developers/sponsors of the intervention.

	1	2	3	4	5	
subitem not at all important	○	○	◉	○	○	essential

Does your paper address subitem X27-i?

Copy and paste relevant sections from the manuscript (include quotes in quotation marks "like this" to indicate direct quotes from your manuscript), or elaborate on this item by providing additional information not in the ms, or briefly explain why the item is not applicable/relevant for your study

Yes

About the CONSORT EHEALTH checklist

As a result of using this checklist, did you make changes in your manuscript? *

- ○ yes, major changes
- ○ yes, minor changes
- ◉ no

What were the most important changes you made as a result of using this checklist?

Not applicable

How much time did you spend on going through the checklist INCLUDING making changes in your manuscript *

60m

As a result of using this checklist, do you think your manuscript has improved? *

- ◉ yes
- ○ no
- ○ Other:

Would you like to become involved in the CONSORT EHEALTH group?

This would involve for example becoming involved in participating in a workshop and writing an "Explanation and Elaboration" document

- (x) yes
- ( ) no
- ( ) Other:

Any other comments or questions on CONSORT EHEALTH

Your answer

STOP - Save this form as PDF before you click submit

To generate a record that you filled in this form, we recommend to generate a PDF of this page (on a Mac, simply select "print" and then select "print as PDF") before you submit it.

When you submit your (revised) paper to JMIR, please upload the PDF as supplementary file.

Don't worry if some text in the textboxes is cut off, as we still have the complete information in our database. Thank you!

Final step: Click submit !

Click submit so we have your answers in our database!

SUBMIT

Never submit passwords through Google Forms.

This content is neither created nor endorsed by Google. Report Abuse - Terms of Service - Additional Terms

Google Forms
